# In silico prediction of HIV-1-host molecular interactions and their directionality

**DOI:** 10.1371/journal.pcbi.1009720

**Published:** 2022-02-08

**Authors:** Haiting Chai, Quan Gu, Joseph Hughes, David L. Robertson

**Affiliations:** MRC-University of Glasgow Centre for Virus Research, Glasgow, United Kingdom; University of California San Diego, UNITED STATES

## Abstract

Human immunodeficiency virus type 1 (HIV-1) continues to be a major cause of disease and premature death. As with all viruses, HIV-1 exploits a host cell to replicate. Improving our understanding of the molecular interactions between virus and human host proteins is crucial for a mechanistic understanding of virus biology, infection and host antiviral activities. This knowledge will potentially permit the identification of host molecules for targeting by drugs with antiviral properties. Here, we propose a data-driven approach for the analysis and prediction of the HIV-1 interacting proteins (VIPs) with a focus on the directionality of the interaction: host-dependency versus antiviral factors. Using support vector machine learning models and features encompassing genetic, proteomic and network properties, our results reveal some significant differences between the VIPs and non-HIV-1 interacting human proteins (non-VIPs). As assessed by comparison with the HIV-1 infection pathway data in the Reactome database (sensitivity > 90%, threshold = 0.5), we demonstrate these models have good generalization properties. We find that the ‘direction’ of the HIV-1-host molecular interactions is also predictable due to different characteristics of ‘forward’/pro-viral versus ‘backward’/pro-host proteins. Additionally, we infer the previously unknown direction of the interactions between HIV-1 and 1351 human host proteins. A web server for performing predictions is available at http://hivpre.cvr.gla.ac.uk/.

## Author summary

Human immunodeficiency virus type 1 (HIV-1) is the cause of acquired immunodeficiency syndrome (AIDS), a disease with no effective cure despite decades of research. A better understanding of the molecular interactions between HIV-1 and human host proteins can facilitate the discovery of potential host targets which may be of great importance for the development of antiviral drugs that go beyond mere control of infection. In this study, we elucidate some host-dependency and antiviral factors that may be helpful to distinguish HIV-1 interacting human proteins (VIPs) from non-HIV-1 interacting human proteins (non-VIPs). We also consider the ‘directionality’ in the HIV-1-host protein-protein interactions, i.e., whether the interaction with the host molecule is in the interest of the virus or part of the anti-viral response. We design a machine learning framework to generate models based on the known information and use them for the classification of VIPs and non-VIPs. Our predictions have the potential to provide refined sets of human host targets aiding in the discovery of novel HIV-1 therapeutics.

## Introduction

Human immunodeficiency virus type 1 (HIV-1) is the cause of acquired immunodeficiency syndrome (AIDS) and constitutes a major cause of human disease and associated comorbidities. Virus infection involves viral molecules exploiting the host cell in order to replicate. The engagement of the HIV-1 envelope glycoprotein and cell-surface receptors, CD4 and either the membrane-spanning C-C motif chemokine receptor 5 (CCR5) or C-X-C motif chemokine receptor 4 (CXCR4), initiates virus attachment and entry into the cell [1–3]. Virus molecules including the HIV-1 regulatory factors (*tat* and *rev*) and accessory proteins (*vpr*, *vif*, *nef*, and *vpu*) ensures viral persistence, replication, dissemination and transmission by modulating the surface and intracellular environment of the infected cell [4–8]. The production of HIV-1 *gag*/*pol* polyproteins is essential for assembly, release and maturation of new virions [9]. Protein-protein interactions (PPIs) between virus and host molecules enable the virus to infect and exploit host cell sub-systems to replicate and persist despite the host immune response [1–3,6–11]. Conversely, there are many human host molecules that function as antiviral factors and are part of the immune response [12–14]. Improving our understanding of these HIV-1-host PPIs can provide insights into the molecular mechanisms underlying virus persistence and pathogenesis. Determining the nature of virus-host interactions [15] is thus of importance for the discovery of potential host inhibitors or targets for antiviral therapeutics [16] exemplified by the CCR5 antagonist maraviroc [17]. Intuitively, there are many more possible drug-targets in the host compared to HIV’s compact genome, which codes for relatively few proteins. To efficiently direct laboratory experiments and make use of rapidly accumulating data in the post-genomic era, the development of efficient *in silico* approaches has become an important area of research focus.

Over the past few years, several computational studies on HIV-1 have characterised attributes of HIV-1 interacting human proteins based on various data, e.g., gene ontology (GO) annotations [18], interaction network profiles [19], disease pathways [20] and post-transcriptional modification profiles [21]. A hierarchical biclustering system has been used [15] to designate HIV-1-host PPIs directionality, polarity and control properties. This research demonstrates how the HIV-1 interacting human proteins (VIPs) can be grouped by related virus-associated perturbations and can be distinguished from the non-HIV-1 interacting ones (non-VIPs). Curation of the extensive experimental literature has permitted an HIV-1-host PPI dataset to be compiled [18]. This can be used for the purpose of modelling and predictions via machine learning algorithms. For example, a random forest (RF) model was constructed by including 35 features for the prediction of HIV-1-host interaction pairs [22]. Further work integrated semi-supervised learning, multi-task learning and neural networks [23]. Subsequently, a biclustering-based approach was applied along with an association rule mining technique [24,25]. Supervised machine learning methods [26,27] have also been implemented using the support vector machine (SVM) algorithm and datasets with different positive-to-negative ratios. Based on the assumption that proteins with similar sequence or structural properties tend to share common interaction partners, studies have also predicted possible HIV-1-host interaction pairs by integrating protein short linear motifs (SLiMs) or protein structure information [28–30].

Owing to the contribution made by computational approaches [31–34], it is possible to obtain a list of potential VIPs with high confidence. However, there are still many improvements that can be made. The majority of the published methods [22–30] are highly dependent on the use of limited types of properties of the interacting HIV-1-host molecules. Some of the defined non-VIPs could be false negatives relative to different HIV-1 proteins [35]. For example, non-*env*-interacting protein cyclin T1 (CCNT1) interacts with HIV-1 during infection as it is targeted by *gag* and *tat* proteins [4,36,37]. The specific nature of the molecular interaction is important for understanding pro-viral interactions versus host antiviral activities. Crudely this can be broken down to the ‘directionality’ of the interaction [15]: ‘forward’/pro-viral versus ‘backward’/pro-host proteins, a prediction task addressed for the first time in this study. Additionally, there also exists a group of human host proteins having both pro-viral and pro-host properties, i.e., are ‘bidirectional’ in nature, for example, CD4 [38,39]. Finally, although the expansion of feature coverage provides a clearer picture for the classification problem, it also induces a series of problems such as feature redundancy [40] and overfitting [41–43].

**Fig 1:**
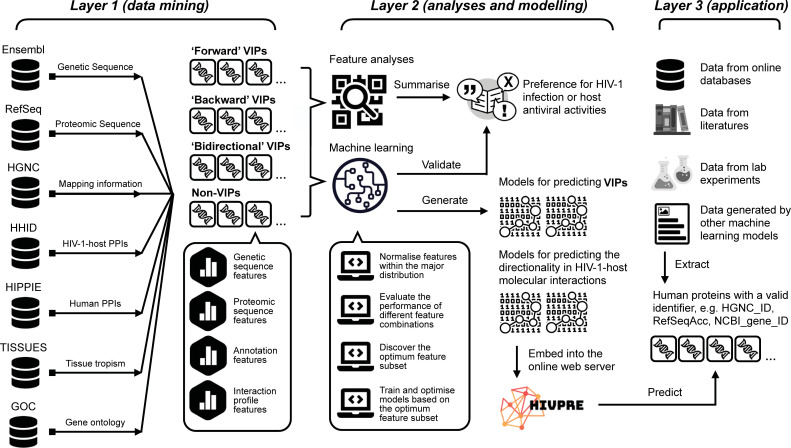
Diagrammatic representation of the project pipeline separated into three procedural layers. The figure is created using images from Wikimedia Commons, https://commons.wikimedia.org. Abbreviations: HIV-1, human immunodeficiency virus type 1; VIPs, HIV-1 interacting human proteins; non-VIPs, non-HIV-1 interacting human proteins; HGNC, HUGO Gene Nomenclature Committee; HHID, the HIV-1 Human Interaction Database; HIPPIE, Human Integrated Protein-Protein Interaction rEference; GOC, Gene Ontology Consortium.

To address these points, we propose a computational approach for the analysis and prediction of HIV-1-host molecular interactions (presented diagrammatically in **Fig 1**). Contrary to previous prediction-based studies [22–30], we introduce a broader definition for the HIV-1 interacting proteins. Human proteins targeting or being targeted by one or multiple HIV-1 proteins are all referred to as VIPs. Non-VIPs represent those human proteins without any record of being directly involved in an HIV-1 interaction. We designed three procedures to maximise the set of the non-VIPs and to reduce their chance of being false negatives. Three tags: ‘forward’ (pro-viral), ‘backward’ (pro-host) and ‘bidirectional’ (pro-viral and pro-host) were assigned to VIPs to capture the direction of the virus-host interaction during the HIV-1 life cycle [15,44]. In total, we encoded 671 features based on the data retrieved from multiple databases [44–50] to characterise the human proteins by genetic, transcriptomic, proteomic and network information. We also measured the contribution of individual features and different feature combinations. We constructed different feature sets via two feature selection schemes to generate prediction models on the training datasets with the SVM method [51]. Performance on the testing datasets demonstrates good prediction quality and generalization capability of our VIP prediction models. A web server for HIV-1-host molecule prediction is available at http://hivpre.cvr.gla.ac.uk/.

## Methods

### Dataset curation

We retrieved 16215 HIV-1-host PPI records from the HIV-1 Human Interaction Database (HHID) (https://www.ncbi.nlm.nih.gov/genome/viruses/retroviruses/hiv-1/interactions/) [44] involving 7120 HIV-1-host interaction pairs and 3854 distinct VIPs (**S1 Data**). Protein sequences for the VIPs were collected from the NCBI’s RefSeq database [46]. To avoid over-representation of similar protein sequences in the dataset, we grouped them into 2881 clusters using CD-HIT [52,53] with a threshold of 40% sequence similarity [52,53], and picked the longest sequence in each cluster as representative. This was to prevent producing feature vectors with high similarity, biasing the prediction performance. These 2881 representative VIPs formed our positives in dataset S1 (**Table 1**).

**Table 1:** Breakdown of VIP and non-VIP datasets used.

Dataset^a^		Positives	Negatives
Main dataset S1		2881 VIPs	7261 non-VIPs
	Training S1’	2304 VIPs	2304 non-VIPs
	Independent testing S1”	577 VIPs	4957 non-VIPs
Main dataset S2		188 backward VIPs	1007 forward VIPs
	Training S2’	150 backward VIPs	150 forward VIPs
	Independent testing S2”	38 backward VIPs	857 forward VIPs
Reference dataset S3		335 bidirectional VIPs	
Blind testing dataset S4		1351 undefined VIPs	
Testing dataset S5		234 VIPs	
Testing dataset S6		356 VIPs	

^a^Dataset S1 and S2 were constructed for the prediction of VIPs and their directionality in the HIV-1-host PPIs. 80% of positives and an equal number of negatives were randomly selected for training while the remaining 20% of proteins were used for testing. Dataset S3 was constructed for prediction of ‘bidirectional’ VIPs while S4 was constructed for the prediction of putative forward, backward or bidirectional VIPs. Testing datasets S5 and S6 were retrieved from two resources with high experimental confidence: the HIV-1 infection pathway in Reactome [60], https://reactome.org/PathwayBrowser/#/R-HSA-162906 and viral host-dependency epistasis map linked to the HIV function [61]. The lists of proteins sampled for training and independent testing are provided in **S2 Data**.

Abbreviations: HIV-1, human immunodeficiency virus type 1; VIPs, HIV-1 interacting human proteins; non-VIPs, non-HIV-1 interacting human proteins.

**Fig 2:**
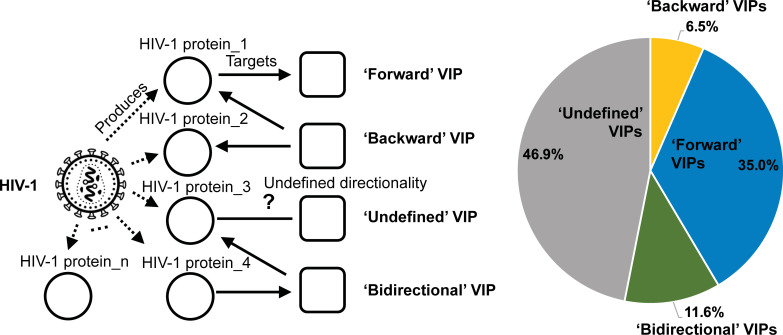
Representation of the characterisation of the types of virus interacting proteins (VIPs). VIPs were tagged as ‘forward’, ‘backward’ or ‘bidirectional’ based on the key words describing their interaction with HIV-1 proteins [44] and directionality designated by MacPherson *et al*. (https://doi.org/10.1371/journal.pcbi.1000863.s004) [15]. The direction was classed as ‘undefined’ if this information is not available. The direction tag for each VIP is provided in **S1 Data**. The figure is created using BioRender, https://biorender.com/. Abbreviations: HIV-1, human immunodeficiency virus type 1; VIP, HIV-1 interacting human protein; PPI, protein-protein interactions.

Following the methods of MacPherson *et al*. [15], we assigned the VIPs direction tags: ‘forward’, ‘backward’ or both/‘bidirectional’ (**Fig 2**). The forward VIPs (e.g., C-X-C motif chemokine ligand 10, CXCL10) are pro-viral proteins. These host molecules are targeted by HIV-1, so-called host-dependency factors [54] and have no recorded antiviral response to the infection during the virus life cycle [55]. The backward VIPs (e.g., apolipoprotein L1, APOL1) are pro-host proteins that are associated with control or inhibition of the viral infection [56]. The bidirectional VIPs (e.g., CXCR4) are targeted by HIV-1 (forward direction) and can produce pro-host responses (backward direction) by influencing the same or different HIV-1 proteins during the viral infection. Some of these are potential therapeutic targets to inhibit virus replication by making host molecules unavailable to the virus [57,58]. Since the ‘direction’ of some HIV-1-host molecular interactions have not been clearly defined, these VIPs were not included in our analysis. Collectively, we obtained 188 (~6.5%) backward, 1007 (~35.0%) forward, 335 bidirectional (~11.6%) and 1351 (~46.9%) undefined VIPs from dataset S1 to construct another three datasets, S2, S3 and S4 for direction-related predictions (**Table 1**).

We performed three procedures to improve the quality of the non-VIP dataset and reduce potential false negatives. First, we chose human proteins produced by the canonical transcript since these proteins are assumed to express the main function of the gene [59]. Second, human proteins sharing more than 40% sequence similarity with any of the reported 3854 VIPs in the HHID were excluded to prevent sequences similar to the known VIPs overly influencing the modelling and predictions. Third, we controlled for the sequence similarity of non-VIPs at a 40% level as we did for VIPs. This is intended to prevent predictions being influenced by similar combinations of feature vectors. As a result, we obtained 7261 nonredundant non-VIPs to form the negatives in dataset S1 (**Table 1**).

As we applied different criteria compared to others to classify our VIPs/non-VIP datasets, it was hard to make direct comparisons between our predictions and previous studies [22–30]. We introduced two testing datasets consisting of VIPs with high experimental confidence from Reactome [60] and Gordon *et al*. [61] in order to assess the generalization capability of our machine learning models. A breakdown of the VIPs and non-VIPs used in this study is listed in **Table 1** and more detailed information is provided in **S2 Data**.

### Feature generation

In this study, we encoded 671 different features mainly from six online databases: Ensembl [47], RefSeq [46], TISSUES [48], Human Integrated Protein-Protein Interaction rEference (HIPPIE) [50], HHID [44] and the Gene Ontology Consortium (GOC) [49]. Among them, 537 features were used to distinguish the VIPs from non-VIPs while 584 features were used to investigate the directionality of the HIV-1-host molecular interactions. Based on the data sources, our encoded features could be divided into four groups: (1) genome-based sequence, (2) proteome-based sequence, (3) annotation-based and (4) interaction profile-based features. The source code for generating these features is available at: https://github.com/HChai01/HIVPRE.

#### Genome-based sequence features

We compiled 107 genome-based sequence features for each human protein which included alternative splicing, nucleotide composition, codon usage and a measure of evolutionary conservation. Information in the alternative splicing data was encoded into four features to represent the evolution of phenotypic complexity in human genes [62,63]: the number of transcripts, protein-coding transcripts, exons and unit exon in transcripts (UET). Nucleotide composition represented the distribution of four basic nucleobases and their phosphodiester bonds-combinations, e.g., CpG, in the coding region of genetic sequences [64]. The usage of the existing 64 codons was calculated in each nucleotide sequence to reflect the balance between mutational biases and natural selection for translational optimization in different classes [65]. For evolutionary conservation, we collected the data from BioMart [47] and calculated the number of paralogues, synonymous substitutions (ds), non-synonymous substitutions (dN) and the ratio of dN to dS within human paralogues and orthologues in four homininae genomes: chimpanzee, gorilla, orangutan and gibbon. These features were used to assess the evolutionary selection pressure acting on the protein sequences [66].

#### Proteome-based sequence features

We encoded 251 features from proteome-based sequence data for the prediction of VIPs and their directionality. Discrete sequence information was calculated as amino acid compositions, while linear information was analysed from the perspective of SLiMs and intrinsic disorder. We generated 37 types of amino acid composition based on the differences in individual amino acids or their physiochemical attributes [67]. Ambiguous or other types of amino acids, e.g., selenocysteine, pyrrolysine etc. were masked as ‘X’ and ignored in this study. We used MERCI [68] to detect conserved sequence patterns as a result of strong purifying selection [69], obtaining 206 motifs representing putative SLiMs overrepresented in the group of VIPs and backward VIPs (Pearson’s Chi-squared test, P<0.05). The occurrence of these potential SLiMs was then split and encoded into 206 non-parametric features with a binary system. Four features measuring the overall representation of VIP- or backward VIP-enriched SLiMs were added as hedges against random error caused by data imbalance [70]. The disordered regions in human protein sequences were identified using Espritz [71] and IUPred [72] as such regions have been linked to VIPs [73].

#### Annotation-based features

We encoded 292 annotation-based features with a binary system from the collected tissue and gene expression data. Among these, 66 features were generated by mapping the GO terms to the child term of three main GO root terms: molecular function (GO:0003674), cellular component (GO:0005575) and biological process (GO:0008150) [49]. They characterise the domain in which human proteins may be involved such as binding (child term of molecular function, GO:0005488), intracellular (child term of cellular component, GO:0005622) and metabolic process (child term of biological process, GO:0008152) when interacting or not interacting with HIV-1 molecules [18]. The remaining 226 features were encoded based on the experimentally verified expression data in TISSUES [48] to reflect the association between tissue tropism and HIV-1 infection at a molecular level [74].

#### Interaction profile-based features

Interaction profile-based features were generated from HIV-1-host PPIs [44] and the human interactome [50]. We used 11 features to represent the degree to which a known VIP was central to the life cycle of HIV-1 [1–3]. Specifically, one feature was encoded to count the number of HIV-1 gene-products interacting with human host molecules and the remaining ten were binary-encoded to capture the interaction relationship between the host molecule and the corresponding HIV-1 gene-product, e.g., *gag*, *tat* or the antisense protein gene *asp* [75]. We retrieved 332,701 experimentally verified human-human PPIs with confidence scores higher than 0.63 involving 17,607 human proteins from HIPPIE [50] to pinpoint proteins with potential pathological or therapeutic relevance [76,77]. NetworkAnalyzer [78] was used to calculate ten different network features including the average shortest distance, degree, neighbourhood connectivity, betweenness, stress, closeness, eccentricity, radiality, topological coefficient and clustering coefficient. Human proteins not involved in the human-human PPI network were assigned zero values for all of the aforementioned network features.

### Supervised machine learning and feature selection

**Fig 3:**
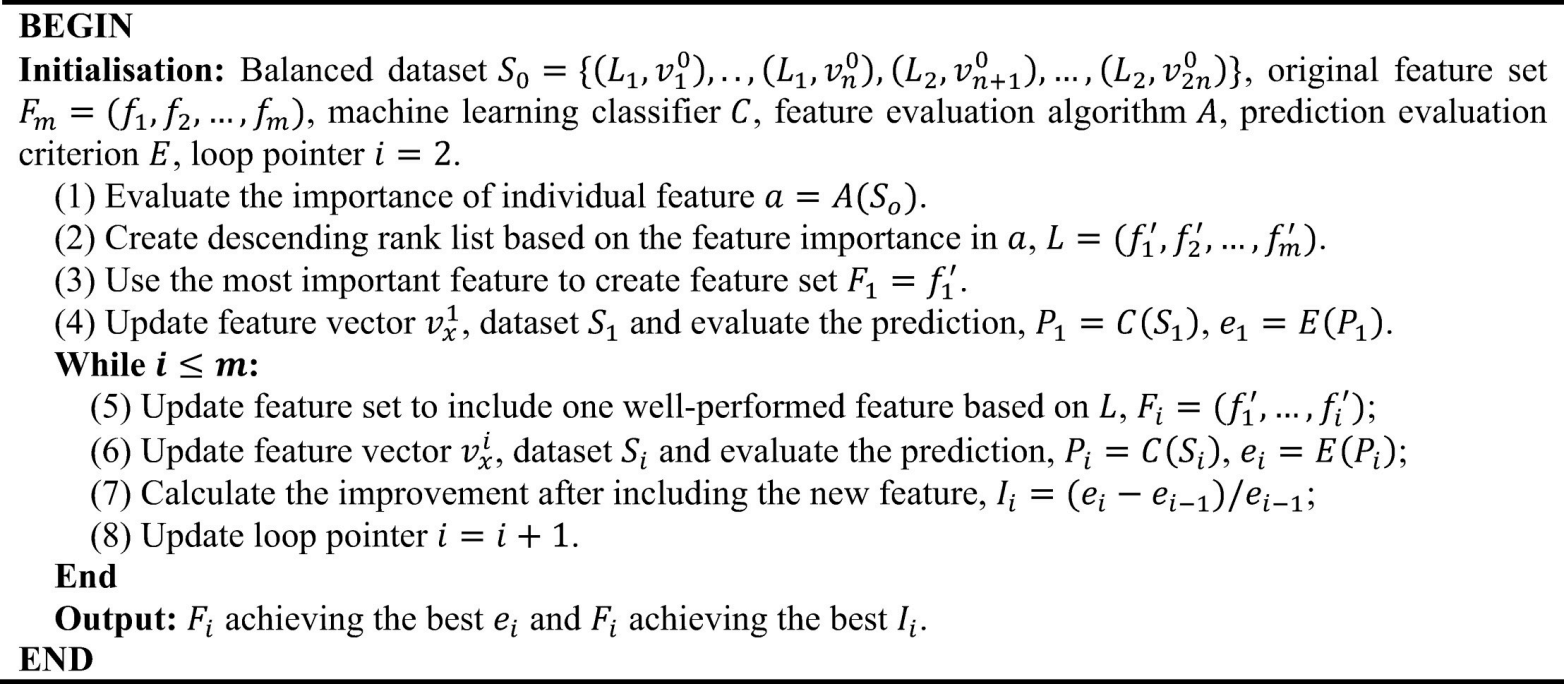
The pseudo-code of the feature selection Scheme 1. We used the SVM model [51] as the base machine learning classifier and the Fisher-Markov Selector [81] to calculate the importance of an individual feature. AUC was chosen as the prime criterion to evaluate the prediction performance on datasets with multiple labels. Abbreviations: SVM, support vector machine; AUC, area under the receiver operating characteristic curve.

We applied a supervised machine learning method for the prediction tasks. We used the SVM model with the radial basis function [51] after comparing it with the k-nearest neighbors (KNN), decision tree (DT) and random forest (RF) algorithms [33]. The SVM algorithm aims to find an appropriate hyperplane in the feature space for classifying the majority of positive and negative samples. It can tolerate the existence of some noisy or incorrect data but may be biased by different feature scales or imbalanced positive-to-negative ratios as it was designed to calculate the margin of the data [79]. Additionally, although the SVM algorithm can map the current feature space to a higher dimensional one for better classification [51], it is a sub-optimal strategy for including too many features for modelling even if they are all instructive. This can result in overfitting of the machine learning model [42] leading to a loss of robustness [80]. To address these points, we first used an undersampling strategy [70] to randomly construct balanced training datasets (**S2 Data**). Second, parametric features were normalised according to their majority distribution in order to share an equal range with non-parametric features:

$$Norm(v)=\left\{ \begin{array}{c} 1,v>\mathrm{UB}\left(v\right)\\ \frac{v-\mathrm{LB}\left(v\right)}{\mathrm{UB}\left(v\right)-\mathrm{LB}\left(v\right)}\\ 0,v<\mathrm{LB}\left(v\right) \end{array}\right.,\mathrm{LB}\left(v\right)<v<\mathrm{UB}\left(v\right)$$ (1)

where *LB*(*v*) and *UB*(*v*) are the lower and upper bound representing the 5^th^ and 95^th^ percentile within the target feature values. Next, we used an SVM-based selection scheme with the evaluation of area under the receiver operating characteristic curve (AUC) to optimise the feature set for the general case (**Fig 3**). In this scheme, we introduced the Fisher-Markov Selector [81] to calculate the importance of an individual feature. We assumed that the usage of better performing features are less likely to negatively influence the complementarity of features in the set, which is crucial to training and modelling [40]. This feature selection scheme produced two outcomes: the optimum feature set and the lowest number of features.

**Fig 4:**
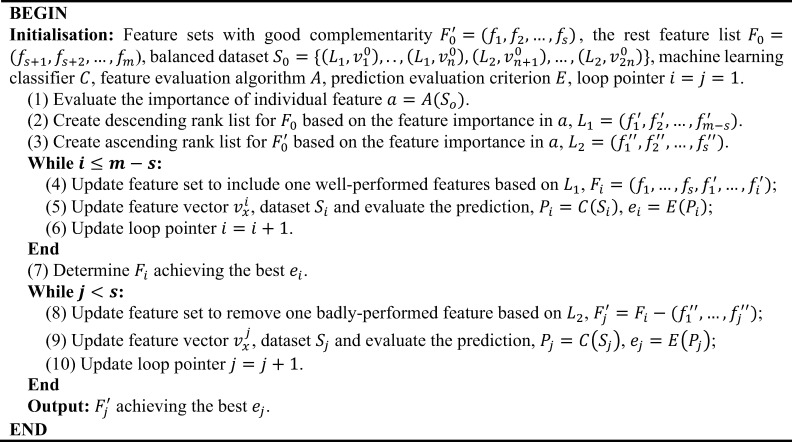
The pseudo-code of the feature selection Scheme 2. We used the SVM model [51] as the base machine learning classifier and the Fisher-Markov Selector [81] to calculate the importance of an individual feature. AUC was chosen as the prime criterion to evaluate the prediction performance on datasets with multiple labels. Abbreviations: SVM, support vector machine; AUC, area under the receiver operating characteristic curve.

The complementarity of different features implies information synergies, which can be measured by calculating the change of system entropy after the introduction of the additional features [40,82,83]. However, it is hard to discriminate if the combination of several random features can achieve better complementarity compared to using an equal number of well performing features. The selection strategy requires reconsideration if the impact of feature synergy has overwhelmed the usage of ‘important’ features on the prediction performance. Here, we use a second feature selection scheme which takes into account both feature importance and complementarity (**Fig 4**). As opposed to the first feature selection scheme (**Fig 3**), this scheme was processed by focusing on a set of features with good complementarity. It contained two main branches: the first expands the coverage of features by introducing well-performing features, while the second reduces the dimension of the feature sets by removing poorly performing features.

### Performance evaluation

In order to assess the performance of different feature subsets, we adopted five-fold cross-validation on training datasets (dataset S1’ and S2’), in which human proteins were randomly divided into five nearly equal parts and further generated five different testing (one portion) and training (the remaining four portions) sets. The overall quality of prediction models constructed from the feature subset was then evaluated based on the produced prediction scores via six criteria including sensitivity, specificity, accuracy, precision, Matthews Correlation Coefficient (MCC) [84] and AUC on the combination of five separate testing results. The evaluation of other independent testing datasets was also processed with the aforementioned six criteria, except in the case of the reference dataset S3, testing dataset S5 and S6, which only used sensitivity controlled by the threshold.

## Results

### HIV-1-host interaction pairs

**Fig 5:**
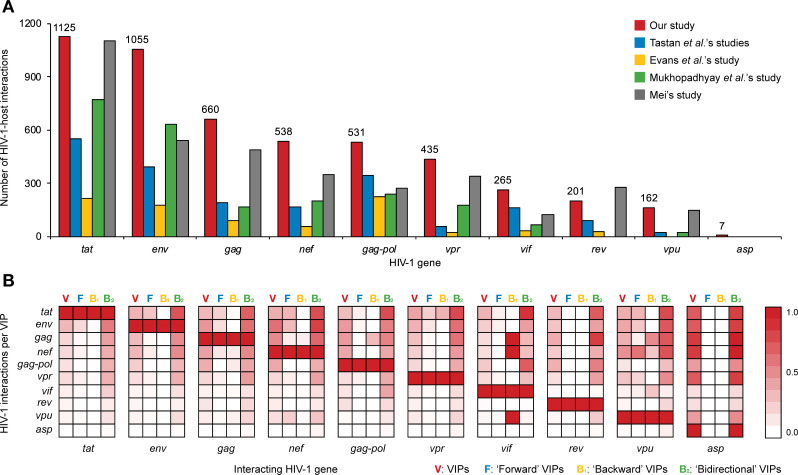
Comparison of HIV-1-host interaction datasets. (A) Comparison of datasets used in previous studies [22–24,27,29]. (B) Illustration of the preference of co-occurring HIV-1-host interactions for the VIPs (**V**), forward VIPs (**F**), backward VIPs (**B**_**1**_) and bidirectional VIPs (**B**_**2**_). Abbreviations: HIV-1, human immunodeficiency virus type 1; VIP, HIV-1 interacting human protein.

Compared with available benchmark datasets [22–24,27,29], our main dataset S1 includes more HIV-1-host PPI data than previous studies (**Fig 5A**). The majority of HIV-1-host molecular interactions are associated with *env*-mediated membrane fusion [1–3] and *tat*-mediated transcellular transport [4,5,85]. In our main dataset S1, there are 996 (~35%) VIPs with interactions with multiple HIV-1 proteins. Some VIPs such as nuclear factor kappa B subunit 1 (NFKB1), interferon gamma (IFNG) and interferon beta 1 (IFNB1) are reported to interact with products produced by almost all HIV-1 genes [86–92]. **Fig 5B** illustrates the preference of co-occurring HIV-1-host PPIs interfering or being induced by the same VIP. It reveals a picture of host targets shared among HIV-1 gene products of *tat*, *env*, *gag*, *nef*, *gag-pol* and *vpr*, and the interaction preference underlying HIV-1 invasion, replication and assembly [1,7,9]. Despite the rank being ordered by the number of HIV-1-host interacting pairs (**Fig 5A**), HIV-1 *tat*-interacting proteins were marginally more frequently connected to *vpr* than *gag-pol*: 0.16 versus 0.14 per VIP, respectively. Interestingly, *tat* was less involved in the interactions with HIV-1 *gag*-, *nef*- and *gag-pol*-interacting proteins than expected. HIV-1 *env*-interacting proteins showed a preference to interact with *nef*, which is also involved in the early stage of the HIV-1 infection [7].

Statistical results indicate an overlap of human host proteins targeted by *env*, *tat* and *nef*. Forward VIPs targeted by HIV-1 *gag-pol*, *vif* and *rev* were less likely to interact with other HIV-1 proteins. An estimated 61% of forward VIPs targeted by *vpu* were also influenced by *nef*. After checking data with more detailed directionality information, we found that the forward or backward VIPs were generally associated with fewer interactions, while the bidirectional VIPs tended to be associated with higher numbers of interactions (**Figs 5B and S1**). Compared with other forward VIPs, *nef*- or *vpu*-interacting forward VIPs tended to be targeted by more HIV-1 proteins (Mann–Whitney U test: P = 3.0E-35). Meanwhile, *vpu*-interacting backward VIPs were more frequently targeted by multiple HIV-1 proteins than other backward VIPs (P = 4.2E-05). Collectively, it is common to observe human host proteins interacting with multiple HIV-1 proteins [44].

### Feature analyses of the compiled data

In this study, we obtained 2881 nonredundant VIPs from 16215 HIV-1-host PPI records and 7261 high-quality non-VIPs from the human proteome. 1530 (~53%) of the VIPs (datasets S2 and S3) showed clear directionality: forward, backward, or bidirectional (**Fig 2**). In total, 671 features were collected from the genetic sequence, proteomic sequence, annotation and interaction profile data. To investigate the predictive signals in our compiled data, we analysed the feature representation in different human proteins.

Our characterisation using evolutionary-related features revealed a consistent pattern linked to HIV-1 infection (**S1 Appendix**). Higher numbers of protein-coding transcripts, duplication rates and evolutionary conservation correlate with the HIV-1-host PPIs. A significantly biased distribution of one-transcript or one-protein-coding-transcript human genes in the VIP versus non-VIP classes provided a strong signal of inhibition associated with the HIV-1 infection (**S1 Appendix**, Pearson’s Chi-squared test: M_1_ = 12.4%, M_2_ = 29.3%, P = 2.8E-71). These results suggest that high conservation has a tendency to be associated with pro-viral interactions, consistent with the documented ancient nature of virus-host interactions [93].

Analyses of the nucleotide and protein sequences were conducted using 354 features. The results indicated that VIPs and non-VIPs had some significant differences in their sequence patterns, e.g., in nucleobase composition linked to putative SLiMs (**S2 Appendix**). For instance, enrichment of adenine, depletion of cytosine and differential codon usage preferences of VIP genes influenced the distribution of amino acids in the protein sequence [94], which also contributed to the signal distinguishing the VIPs from non-VIPs. 85 SLiMs were detected to be more enriched in VIP sequences than in non-VIP sequences (Pearson’s Chi-squared test: P<0.05). Co-occurrence of these putative SLiMs showed a cumulative effect resulting in better classifying of VIPs versus non-VIPs. Pro-viral and pro-host signatures of the VIPs were also reflected in sequence patterns and by intrinsic disorder status in the protein sequence. These results demonstrate the differential representation of sequence-based features provides a useful signal for prediction purposes.

Distinct from the aforementioned evolutionary-related and sequence-based features, annotation-based features are more straightforward with direct functional relevance [95]. Analyses of GO profiles revealed that the VIPs were more involved in cellular process (GO:0009987), binding (GO:0005488) and had a stronger association with organelles in the host cell (GO:0043226) than non-VIPs (Pearson’s Chi-squared test: P = 9.9E-118, 1.0E-84 and 2.0E-70, respectively) (**S3 Appendix**). Within the group of VIPs, the bidirectional VIPs were highlighted for their prevalent response to stimulus (GO:0050896) and frequent involvement in biological regulation (GO:0065007) (**S3 Data**). Analyses of tissue tropisms indicated that the VIPs were more likely to be found in heart- or hematopoietic system-related tissue (**S3 Appendix**). Compared with the forward VIPs, the backward VIPs were less involved in the hematopoietic system but were more expressed in brain-related tissues, such as the brain stem and cerebral lobe (**S3 Data**). Cells originating from stem cells and differentiating in lymphoid tissues were favoured by backward VIPs and the relationship between backward VIPs and CD8+-presenting cells was evident, showing a clear relationship between HIV-1 infection and the host antiviral immune responses [96,97]. In view of the nature of the HIV-1-host molecular interactions, these function-related features are anticipated to perform well in the prediction tasks. However, they may not represent an optimum property in machine learning tasks due to gaps in annotations.

### Performance of different feature sets in the training stage

#### Models for predicting the VIPs

In this study, we encoded 537 features for the prediction of the VIPs. According to the data source from which they were extracted, we divided these features into four categories: genome-based, proteome-based, annotation-based and interaction profile-based features. We first tested the performance of features in different categories on the balanced training datasets and found that annotation-based features performed the best, achieving the highest AUC value at 0.8090 on dataset S1’ (**Table 2**). On the same dataset, the combination of interaction profile-based features produced some good predictions even if only 10 features were included. However, the performance of proteome-based features was poor on dataset S1’. By combining all of the encoded 537 features, we found the classifier could produce a better performance (AUC = 0.8324) than using features by individual categories (AUC = 0.7118, 0.6641, 0.8090, 0.7487, respectively) (**Table 2**). We compared the SVM with another three machine learning models: KNN, DT and RF [33]. We used the square root of the size of the training samples as the k-value for the KNN algorithm [98]. We found this algorithm was biased to the positive class and did not achieve a better prediction performance than the SVM model. The DT algorithm was designed with a feature selection scheme, which helped it to better split the dataset for lower system entropy [82]. It used 278 out of 537 features and had the worst performance among the different machine learning algorithms compared. We initialised the RF algorithm with 50 trees and repeated the modelling process ten times to balance bootstrapping of the dataset and selection of features [99]. The prediction performance of the RF algorithm on S1’ was promising but did not surpass that of the SVM model. These results suggest that the majority of features encoded for the prediction of the VIPs are contributing to the signal, but the complete feature set is not optimal for reliable prediction since it includes some poorly performing features.

**Table 2:** The performance of different feature sets on the training datasets over five-cross validations.

Dataset^a^	Algorithm	Features	Features number	Threshold^e^	Sensitivity	Specificity	Accuracy	MCC	AUC
S1’	SVM	Genetic sequences	107	0.51	0.613	0.700	0.656	0.314	0.7118
	SVM	Proteomic sequences	128	0.51	0.595	0.649	0.622	0.244	0.6641
	SVM	Annotations	292	0.57	0.663	0.806	0.735	0.475	0.8090
	SVM	Interaction profiles	10	0.52	0.611	0.777	0.694	0.394	0.7487
	SVM	Combination	537	0.56	0.690	0.817	0.754	0.512	0.8324
	KNN^b^	Combination	537	0.35~0.39	0.766	0.633	0.699	0.402	0.7772
	DT^c^	Partial	278	N/A	0.633	0.642	0.637	0.275	N/A
	RF^d^	Random	Random	0.44~0.52	0.733±0.035	0.752±0.030	0.742±0.004	0.486±0.009	0.8157±0.0031
	SVM	Top-ranked 33	33	0.54	0.645	0.718	0.681	0.363	0.7468
	SVM	Top-ranked 193	193	0.48	0.748	0.751	0.750	0.499	0.8261
	KNN^b^	Optimum	441	0.43~0.48	0.689	0.720	0.705	0.410	0.7734
	SVM	Optimum	441	0.52	0.727	0.787	0.757	0.514	0.8344
S2’	SVM	Genetic sequences	107	N/A^f^	N/A^f^	N/A^f^	N/A^f^	N/A^f^	N/A^f^
	SVM	Proteomic sequences	164	0.40	0.860	0.633	0.747	0.507	0.8023
	SVM	Annotations	292	0.46	0.767	0.520	0.643	0.296	0.6786
	SVM	Interaction profiles	21	0.51	0.740	0.633	0.687	0.375	0.7108
	SVM	Combination	584	0.46	0.807	0.553	0.680	0.372	0.7383
	KNN^b^	Combination	584	0.50~0.54	0.400	0.833	0.617	0.259	0.6501
	DT^c^	Partial	66	N/A	0.673	0.660	0.667	0.333	N/A
	RF^d^	Random	Random	0.38~0.58	0.706±0.134	0.710±0.167	0.708±0.030	0.432±0.045	0.7609±0.0270
	KNN^b^	Optimum	129	0.27~0.36	0.487	0.873	0.680	0.390	0.7509
	SVM	Optimum	129	0.44	0.853	0.680	0.767	0.542	0.8260

^a^Dataset S1’ and S2’ were balanced training datasets constructed via an undersampling strategy [70] from dataset S1 and S2, respectively (**Table 1**). Compositions of these two datasets are provided in **S2 Data**.

^b^k-value here was determined as the square root of the size of the training samples in the five-fold cross validation

^c^the DT algorithm selected 278 and 66 features from the original feature sets for the two modelling tasks

^d^the RF algorithm used 50 randomly grown trees and the modelling and validation procedures were repeated 10 times

^e^threshold was set by maximizing the value of MCC

^f^‘N/A’ was denoted if the prediction quality of the generated classifier was worse than a random guess.

Abbreviations: SVM, support vector machine; KNN, k-nearest neighbors; DT, decision tree; RF, random forest; MCC, Matthews Correlation Coefficient; AUC, area under the receiver operating characteristic curve.

**Fig 6:**
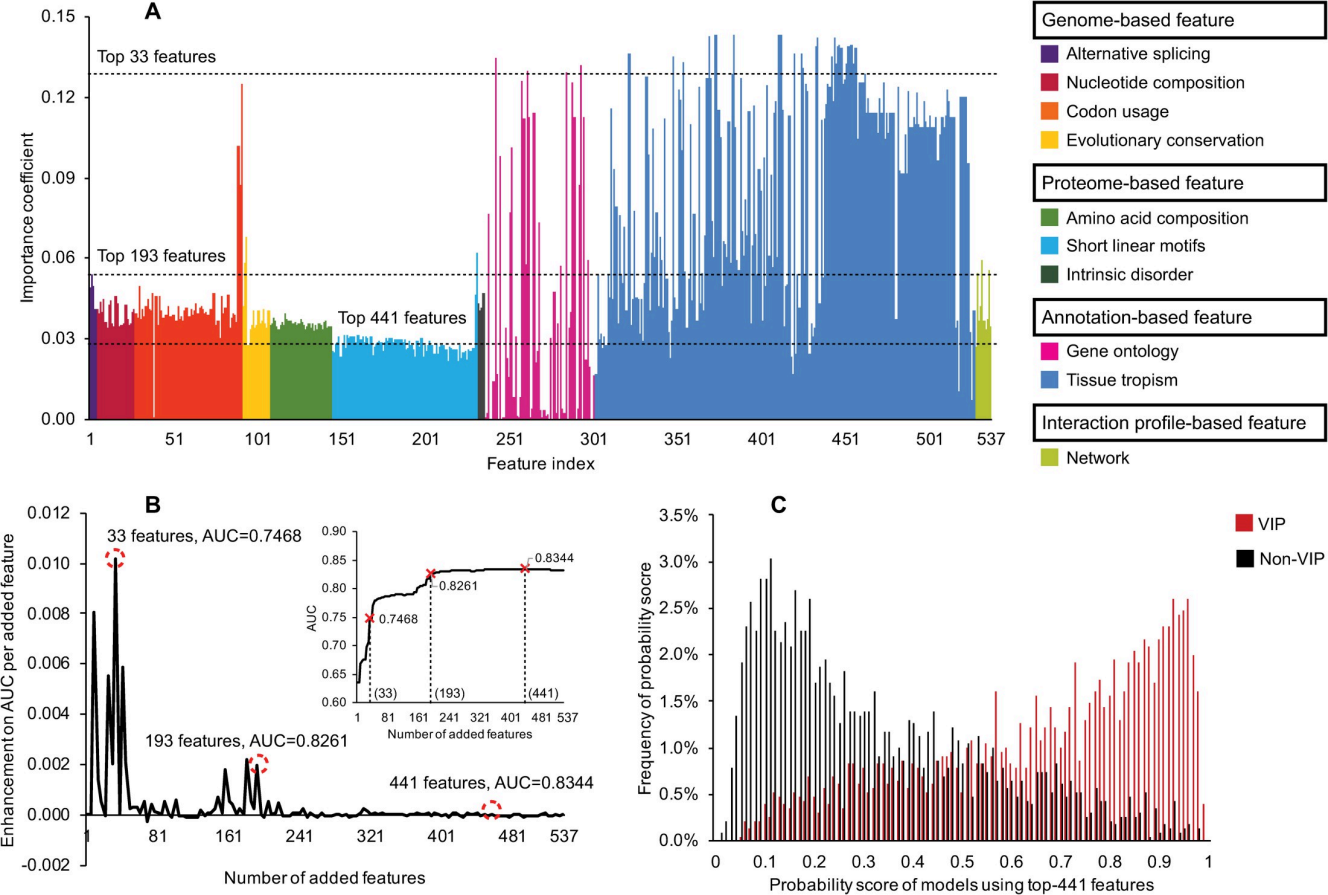
The performance of different features for the prediction of VIPs. (A) The importance of different features. (B) Enhancement the prediction performance by adding more features. (C) The distribution of prediction scores (for VIPs and non-VIPs) generated by model using the top 441 features. In (A) the importance of an individual feature was recorded by averaging the results on the balanced training datasets generated by ten-round undersampling procedures [70] on dataset S1. The ranked list of the encoded 537 features is provided in **S4 Data**. Abbreviations: HIV-1, human immunodeficiency virus type 1; VIPs, HIV-1 interacting human proteins; non-VIPs, non-HIV-1 interacting human proteins; AUC, area under the receiver operating characteristic curve.

In order to find a better feature subset for the prediction of the VIPs, we first used the Fisher-Markov Selector [81] to calculate the importance of individual features (**Fig 6A**). The results demonstrated the importance of gene ontology (e.g., involvement in metabolic process, ranked 24^th^) and tissue tropism features for prediction (e.g., expression in monocyte, ranked 1^st^), even if they were used individually. Based on this ranking of individual features, we then used our first feature selection strategy (**Fig 3**) and five-cross validations to optimise the prediction model. **Fig 6** shows that the classifier obtained reasonable prediction with fairly low numbers of features, e.g., the top 33 features (**S2A Fig**) are near the first inflection point (**Fig 6B)**, while the subset of 193 features were considered optimal as they achieved decent prediction performance (AUC = 0.8261, **S2B Fig**) and there will be less issues with missing data or errors in annotations [100]. The subset of 441 features maximised the prediction performance and are comprised of 105 genome-based features, 84 proteome-based features, 243 annotation-based features and nine interaction profile-based features. The distribution of prediction scores for VIPs and non-VIPs was negatively and positively skewed, with most values clustered around the right and left tails of the distribution, respectively (**Fig 6C**).

#### Models for predicting the direction of the HIV-1-host PPIs

**Fig 7:**
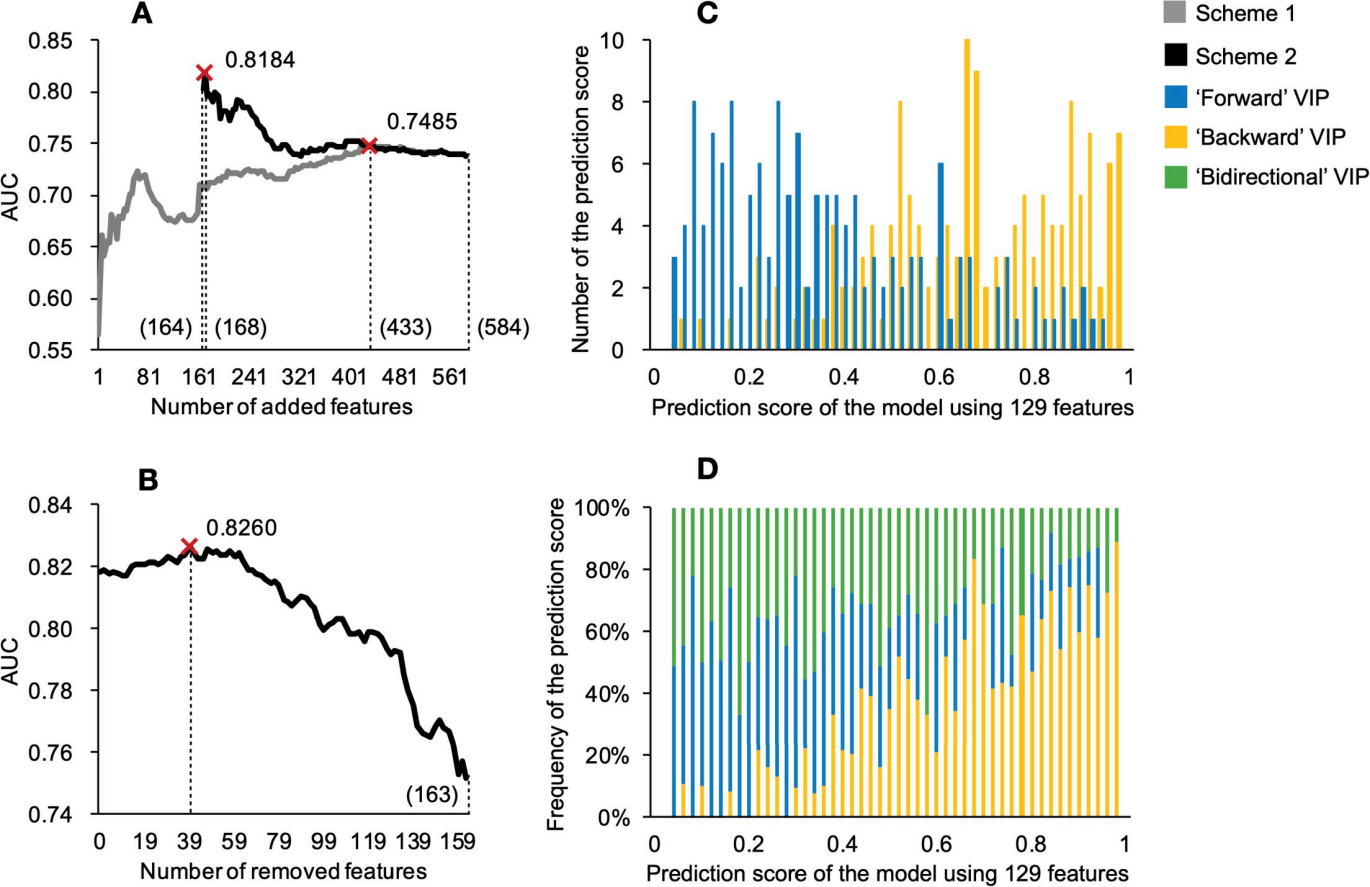
The performance of different features for predicting the backward and forward VIPs. (A) AUC values for increasing numbers of features. (B) AUC values for decreasing numbers of proteome-based features. (C) The counts of prediction scores (for pro-viral/forward VIPs and pro-host/backward VIPs) generated by model using 129 optimum features. (D) The percentage of forward, backward and bidirectional VIPs within different regions of prediction scores (scale = 0.02). Abbreviations: HIV-1, human immunodeficiency virus type 1; VIP, HIV-1 interacting human protein; AUC, area under the receiver operating characteristic curve.

We encoded 584 features for the VIPs to predict pro-host versus pro-virus directionality in the HIV-1-host molecular interactions. Predictions on the training dataset S2’ were different from those on dataset S1’. The performance of genome-based features was even worse than a random prediction (**Table 2**). The combination of proteomic features produced a highly predictive model. The performance of annotation features was not as good as anticipated and the combination of all features made the classifier worse than only using proteomic features. This indicates a big difference between the two prediction tasks highlighted in this study. After checking the importance of features with the Fisher-Markov Selector [81] we found the difference between the generated importance scores was not obvious (**S3 Fig and S4 Data**), which meant the contribution of individual features to the prediction model had not changed appreciably. The comparison of results from the different machine learning algorithms demonstrated that the SVM classifier still worked on dataset S2’ (**Table 2**). These results suggest that the overall complementarity of these 584 features is not as good as those used for predicting the VIPs. There may be a large number of noisy features involved in the complete set, which suppresses the performance of some feature combinations when using our first selection strategy (**Fig 7A**).

Thus, we applied our second feature selection strategy to optimise the prediction model (**Fig 4**). We assumed that the proteomic features might be an ideal set with good complementarity for the initialization as they were better-performing than features in the other categories (**Table 2**). We found performance of the classifier was enhanced, however, it started to decrease after adding four non-proteomic sequence features. We, thus, removed the poorly-performing features in the proteomic sequence feature set. We identified an optimum model generated by 129 features (**Fig 7B**). In that feature subset, 36 amino acid composition, 85 SLiM, four intrinsic disorder, two gene ontology and two tissue tropism features were included (**S4 Data**). Compared with the model generated from the complete feature set, the model using 129 optimum features enhanced the performance by more than 10% from the perspective of the AUC (**Table 2**). Likewise, on the training dataset S2’, the SVM was still superior to the KNN, DT and RF algorithms. Additionally, the model generated from all proteomic features was also recommended as it only required the information from the protein sequence to make reasonable predictions (**S4 Fig**).

Interestingly, testing on the reference dataset S3 suggests that the bidirectional VIPs are closer in properties to the forward VIPs than to the backward VIPs. The forward VIPs may be ‘responding’ to the HIV-1 infection and target HIV-1, making them ‘bidirectional’ [101]. The backward VIPs are less likely to be targeted by HIV-1 so their chances of becoming ‘bidirectional’ are relatively low. The recommended direction based on the prediction score generated by the model using 129 optimum features is listed in **S1 Table**. We could confidently label 60% of the generated VIPs based on the prediction scores as backward, forward, or bidirectional. For prediction scores located in specific ranges, our confidence on the direction of the HIV-1-host molecular interactions could reach as high as 89%.

### Performance on the testing datasets

In this study, we produced three models with the top-33, top-193 and top-441 features on the whole training dataset S1’ for the prediction of VIPs, namely PreVIP-33, PreVIP-193 and PreVIP-441, respectively. Independent testing datasets prepared to assess the generalization capability of these three models was derived from our main dataset S1 through an undersampling strategy [70]. They consist of a random set of 577 VIPs and 4957 non-VIPs. The imbalance ratio of positives (VIPs) to negatives (non-VIPs) in this testing dataset is close to 1:8. PreVIP-33 could successfully predict 40.9% of VIPs and 88.1% of non-VIPs under a threshold of 0.73. The corresponding AUC value of PreVIP-33 was 0.7323. Under the same threshold, the sensitivity and specificity of PreVIP-193 increased to 45.6% and 91.2%, respectively. The optimum threshold for PreVIP-193 was 0.82, under which 34.7% of the VIPs and more than 95% of the non-VIPs were correctly predicted. Among the generated three models, PreVIP-441 achieved the best performance with an AUC value of 0.8079, with the performance of PreVIP-193 close to this (AUC of 0.8034) (**Table 3**). In contrast, PreVIP-33 did not perform well on the testing dataset S1”. When attempting to successfully predict more than half of the VIPs, the ratios of false positives produced by PreVIP-441, PreVIP-193 and PreVIP-33 were 10%, 11% and 19%, respectively.

**Table 3:** The performance of features with different categories on the testing datasets.

Dataset	Model	Feature source	Threshold^a^	Sensitivity	Specificity	Accuracy	Precision	MCC	AUC
S1”	PreVIP-33	Annotation	0.73	0.409	0.881	0.832	0.285	0.248	0.7323
	PreVIP-193	Multiple	0.82	0.347	0.959	0.895	0.495	0.359	0.8034
	PreVIP-441	Multiple	0.73	0.492	0.911	0.867	0.391	0.365	0.8079
S2”	PreDIR-164	Proteomic sequence	0.53	0.658	0.762	0.758	0.109	0.194	0.7110
	PreDIR-129	Multiple	0.70	0.474	0.873	0.856	0.142	0.200	0.7057
S5^b^	PreVIP-193	Multiple	0.82	Sensitivity = 0.577					
	PreVIP-193	Multiple	0.50	Sensitivity = 0.906					
	PreVIP-441	Multiple	0.73	Sensitivity = 0.701					
	PreVIP-441	Multiple	0.50	Sensitivity = 0.910					
S6^b^	PreVIP-193	Multiple	0.82	Sensitivity = 0.416					
	PreVIP-193	Multiple	0.50	Sensitivity = 0.806					
	PreVIP-441	Multiple	0.73	Sensitivity = 0.596					
	PreVIP-441	Multiple	0.50	Sensitivity = 0.817					

^a^thresholds on S1” and S2” were set by maximizing the value of MCC. On testing dataset S5 and S6, two thresholds, i.e., 0.82 and 0.73 were set according to the best performance of PreVIP-193 and PreVIP-441 on testing dataset S1”. In addition, a neutral threshold (0.5) was added for crude assessments.

^b^prediction results on testing dataset S5 and S6 are provided in **S5 Data**.

Abbreviations: MCC, Matthews Correlation Coefficient; AUC, area under the receiver operating characteristic curve; HIV-1, human immunodeficiency virus type 1; VIPs, HIV-1 interacting human proteins; PreVIP-33, machine learning model generated from training dataset S1’ with the top 33 features for the VIP prediction task; PreVIP-193, machine learning model generated from training dataset S1’ with the top 193 features for the VIP prediction task; PreVIP-441, machine learning model generated from training dataset S1’ with the optimum 441 features for the VIP prediction task; PreDIR-164, machine learning model generated from training dataset S2’ with 164 proteome-based features for the directionality prediction task; PreDIR-129, machine learning model generated from training dataset S2’ with the optimum 129 features for the directionality prediction task.

As for predicting the direction of the HIV-1-host molecular interactions, we generated two models with the optimal 129 features and the overall 164 proteomic sequence features on the whole training dataset S2’, namely PreDIR-129 and PreDIR-164. An independent testing dataset prepared to assess the generalization capability of these two models was derived from our main dataset S2 using an undersampling strategy [70]. It is comprised of a random 38 VIPs and 857 non-VIPs. The imbalance ratio of positives (backward VIPs) to negatives (forward VIPs) in this testing dataset is close to 1:22. Compared with PreDIR-129, PreDIR-164 was generally a bit more powerful for achieving higher AUC, at 0.7110 (**Table 3**). The optimum threshold for PreDIR-129 was 0.70, under which 47.4% of the backward VIPs and 87.3% of the forward VIPs were successfully predicted. By contrast, to successfully filter the same number of negatives, PreDIR-164 produced three more false negatives, which showed its drawback in recognising forward VIPs.

**Fig 8:**
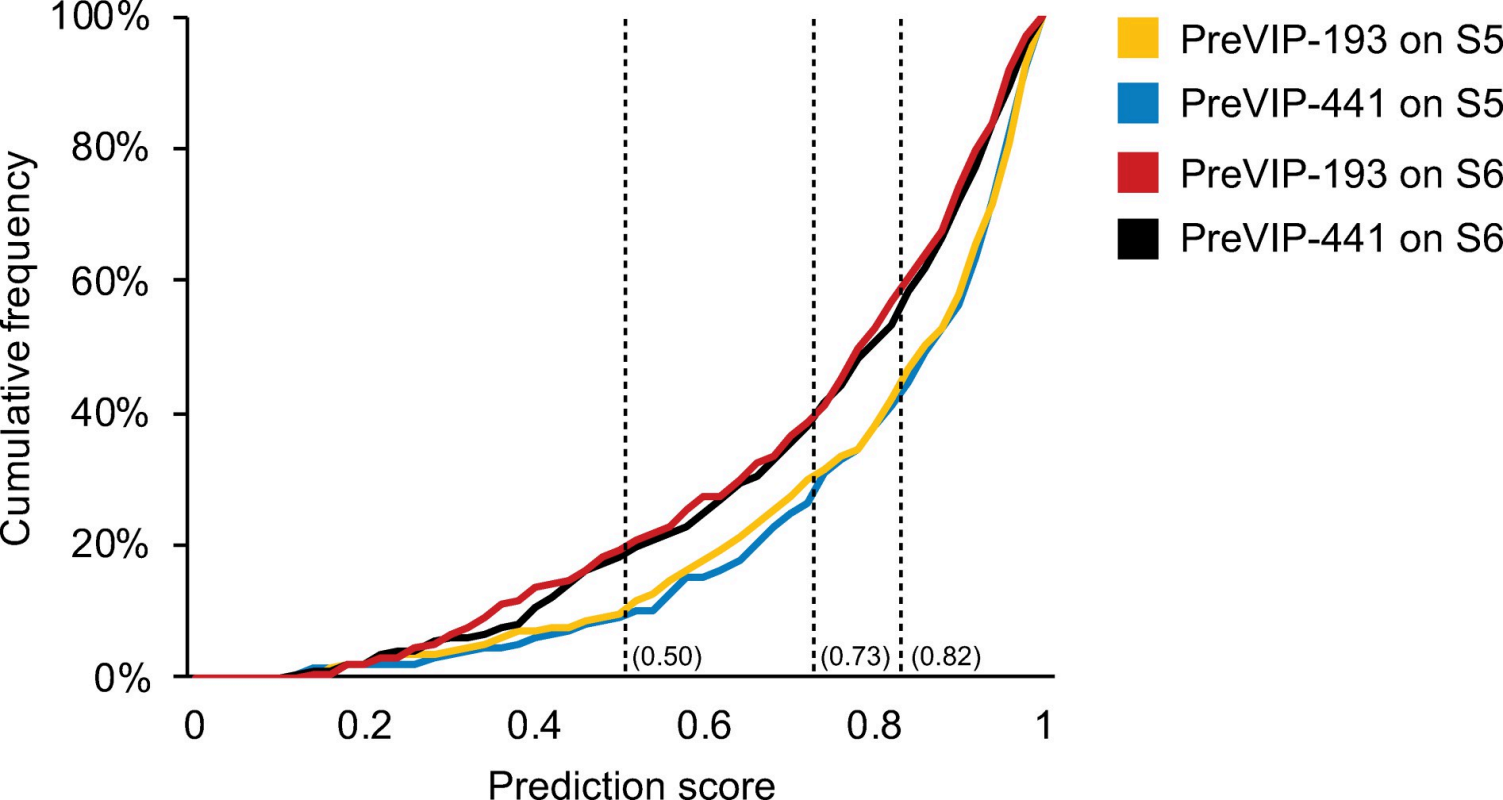
Cumulative distribution of prediction probabilities on the testing datasets S5 and S6. Dataset S5 and S6 were retrieved from Reactome [60] and Gordon *et al*.’s study [61] for the purpose of predicting VIPs. The composition of dataset S5 and S6 is provided in the **S2 Data** file. Prediction results on testing dataset S5 and S6 are provided in **S5 Data**. Abbreviations: VIPs, HIV-1 interacting human proteins; PreVIP-193, machine learning model generated from training dataset S1’ with the top 193 features for the VIP prediction task; PreVIP-441, machine learning model generated from training dataset S1’ with the optimum 441 features for the VIP prediction task.

To further assess the quality of PreVIP-193 and PreVIP-441, we introduced two testing datasets from the HIV-1 infection pathway in Reactome [60] (dataset S5) and an HIV-1 host-dependency epistasis map [61] (dataset S6). **Fig 8** shows that our prediction models performed well in recognising VIPs confirmed with experimental evidence. On testing dataset S5, PreVIP-441 could recognise 70.1% of VIPs under a threshold of 0.73, about 40% more than its expected performance (**Table 3**). It was also capable of successfully predicting more than 90% of VIPs when using a threshold of 0.5. Under the same threshold, PreVIP-193 achieved a similar performance as PreVIP-441. The improvement under the threshold of 0.82 reached as high as 66% when compared with its expected sensitivity (34.7%). Thus, these results demonstrate good generalization capabilities of our models on predicting VIPs involved in the host sub-systems hijacked during HIV-1 infection [60]. Their performance on the testing dataset S6 was also promising with an estimated 20% improvement. The prediction results of PreVIP-193 and PreVIP-441 on testing datasets S5 and S6 are shown in **S5 Data**.

On the blind testing dataset S4, we used PreDIR-129 to predict the direction tag for 1351 ‘Undefined’ VIPs (**Fig 2**). According to known information about potential direction (**S1 Data**) and the recommendation stated in **S1 Table**, 511, 540 and 300 undefined VIPs were predicted as backward, forward and bidirectional, respectively (**S6 Data**). The prediction scores for the putative different VIPs showed significant differences in the majority of regions (**S5 Fig**) (Mann–Whitney U test: P = 2.5E-124, 2.0E-43, 4.9E-19, respectively). 66 of the undefined VIPs had a high probability of being ‘backward’ interactions and in terms of function the literature shows connections to brain-related diseases such as autosomal recessive neurodevelopmental disorder deficiency [102] and Huntington disease [103] (**S2 Table**). 63 of the undefined VIPs were very likely to be ‘forward’ interactions and were involved in some immune system pathways (**S2 Table**). 50 of the undefined VIPs showed strong signals of being ‘bidirectional’ interactions. Interestingly, they appear to be targets of other viruses like human papillomavirus [104] and hepatitis virus [105] (**S2 Table**).

## Discussion

In this study, we propose an *in silico* approach to investigate HIV-1-host molecular interactions with a focus on prediction of the directionality of the virus-host interaction. We used the detailed curation of the biological nature of known HIV-1-host interactions in the HHID [44] to partition interactions as those required by the virus to manipulate the host molecular sub-systems versus host responses to virus infection. Using this dataset, we design a predictive system in which human proteins can be quickly evaluated for their potential to target host (a host-dependency factor), be targeted (the antiviral response), or both (bidirectional interactions). A web server is available at http://hivpre.cvr.gla.ac.uk/. It supports six different identifiers for over 80000 human peptides and can carry out 1000 predictions in less than 15 seconds.

In previous studies [22–30], VIPs were usually labeled based on their interacting HIV-1 status only. According to the data we retrieved from the HHID [44], 1467 out of 3854 human proteins, including some key receptors (e.g., CD4 and CCR5), have interactions with protein products of different HIV-1 genes (**S1 Data**). Such multi-target issues can be accommodated by integrating information on the molecular interactions between human proteins and the HIV-1 interaction type. Previously published prediction-based papers [22–24,26–30] have not accounted for the direction of the HIV-1-host molecular interaction. By contrast, our consideration of the interaction direction contributes to a better understanding of the HIV-1-host interactions and the discovery of potential drug targets [106].

Analysing evolutionary-related information in the transcriptomic and genomics data (**S1 Appendix**), we found HIV-1 was more likely to interact with human proteins encoded from genes with higher numbers of protein-coding transcripts, higher duplication rates and more evolutionary conserved. Presumably this is at least partly because the evolutionary rates for duplicate genes have a tendency to be negatively correlated with the number of paralogues [107], and virus-interacting molecules are often relatively evolutionary ancient [93]. We discovered 85 VIP-enriched putative SLiMs and 121 backward VIP-enriched SliMs from the proteomic sequence data (**S2 Appendix**). We hypothesise that there are some motifs in the sequence of VIPs mediating molecular interactions, making them more likely to target or be the target of HIV-1. Human proteins with longer sequences have a higher probability of including some predictive sequence patterns than those with short sequences. For example, there are over 14000 residues in the sequence of a non-VIP, namely mucin 16 (MUC16), but only 17 VIP-enriched SliMs were observed. However, this signal needs to be treated with caution especially when large numbers of VIP-enriched and backward VIP-enriched SliMs are both detected in the same non-VIP sequence, e.g., midasin (MDN1) (n = 41 and 57). Such ‘non-VIPs’ may potentially be false negatives if some of their SLiM-enriched regions could interact with HIV-1 [108]. We obtained 225 experimentally verified tissue entries from the TISSUES database [48] but found some non-independence of features due to the hierarchical nature of this type of data(**S3 Appendix**). Nonetheless, the annotation data of tissue tropisms was sufficient for distinguishing VIPs from non-VIPs (**S6 Fig**). The later analysis also demonstrated the practical effectiveness of considering these features individually or in combination (**Fig 6A** and **Table 2**).

After finishing all prediction tasks, we assumed that some false negatives were still included in our dataset since we found some of the testing non-VIPs obtained very high prediction scores (**S3 Table**). Based on the testing result given by PreVIP-441 and PreVIP-193, 16 labelled non-VIPs might actually interact with HIV-1 proteins. For example, we found that adapter molecule crk (CRK), TGF-beta-activated kinase 1 and MAP3K7-binding protein 1 (TAB1) and interleukin-1 receptor-associated kinase 4 (IRAK4) are involved in the HIV-1 infection in the Kyoto Encyclopedia of Genes and Genomes (KEGG) database [109] but were not included in the HHID [44]. This provided further support for the predictive value of our machine learning approach. Some features of these human proteins also hinted at their possible roles as VIPs. For instance, alpha-synuclein (SNCA) had a high number of polymorphisms, contained 15 VIP-enriched SLiMs within its 140-length proteomic sequence, expressed in many VIP-preferred tissues and was highly connected with a degree of 168 in our constructed network [50]. As for the prediction of the interaction directionality, some results in **S1 Table** might be ambiguous when being used individually but higher confidence could be obtained when combining the information on known interaction directionality in **S1 Data**. For instance, elongin-B (ELOB) had a prediction score of 0.14 from PreDIR-129 so was initially predicted to be a forward VIP (**S1 Table)**. However, since we found 18 records on the molecular interactions between ELOB and HIV-1 proteins and some outcomes of the interactions showed the clear direction of ‘backward’, ELOB is probably ‘bidirectional’ rather than only ‘forward’ acting.

In conclusion, reliably predicting HIV-1-host molecular interactions is a difficult task and to improve requires a better framework for understanding the nuances of the virus-host relationship. Here we have introduced the directionality of the interaction to this task and demonstrated that there is a predictive signal embedded in the different types of molecules. Many of the features used, however, are only superficially capturing the information embedded in the molecules involved. We are confident that better training datasets and continued development of feature representation of molecules, for example, integrating protein structure and molecular interaction data, will lead to improved predictions in the near future.

## Supporting information

S1 Appendix Characterisation of features linked to alternative splicing and evolution. (PDF)

S2 Appendix Characterisation of features in different sequence patterns. (PDF)

S3 Appendix Characterisation of features in annotation and network profiles. (PDF)

S1 Fig The preference of co-occurring HIV-1-host interactions for different VIPs. Boxes in the plot represent the major distribution of values (from the first to the third quartile); outliers were added for values higher than two-fold of the third quartile; the cross symbol marks the position of the average value including the outliers; upper and lower whiskers showed the maximum and minimum values excluding the outliers. Abbreviations: HIV-1, human immunodeficiency virus type 1; VIP, HIV-1 interacting human protein.
(PDF)

S2 Fig Prediction score generated by models using (A) the top-33 and (B) top-193 features on dataset S1’ over five-cross validation. Abbreviations: HIV-1, human immunodeficiency virus type 1; VIPs, HIV-1 interacting human proteins; non-VIPs, non-HIV-1 interacting human proteins.
(PDF)

S3 Fig Importance of individual features for predicting the backward and forward VIPs. Abbreviations: The importance score of individual features is recorded by averaging the results on the balanced training datasets generated by ten-round undersampling procedures on dataset S2. HIV-1, human immunodeficiency virus type 1; VIPs, HIV-1 interacting human proteins.
(PDF)

S4 Fig Prediction score generated by models using proteomic features on dataset S2’ over five-cross validation. Abbreviations: HIV-1, human immunodeficiency virus type 1; VIPs, HIV-1 interacting human proteins.
(PDF)

S5 Fig Prediction score generated by PreDIR-129 on the blind testing dataset S4. Abbreviations: HIV-1, human immunodeficiency virus type 1; VIPs, HIV-1 interacting human proteins.
(PDF)

S6 Fig Tissue tropisms for different human proteins. Abbreviations: HIV-1, human immunodeficiency virus type 1; VIPs, HIV-1 interacting human proteins; non-VIPs, non-HIV-1 interacting human proteins.
(PDF)

S1 Table The recommended interaction direction for models using the 129 optimum feature set. (PDF)

S2 Table Undefined VIPs with very high probabilities indicating their directionality in the HIV-1-host molecular interactions. (XLSX)

S3 Table Non-VIPs with high prediction scores (>0.95) generated by PreVIP-441 and PreVIP-193. (XLSX)

S1 Data The list of HIV-1 interacting proteins. (TXT)

S2 Data The list of proteins sampled for training and testing. (TXT)

S3 Data Statistical data supporting results in the manuscript and appendix. (XLSX)

S4 Data The importance and usage of features in different machine learning models. (TXT)

S5 Data Prediction results on dataset S5 and S6. (TXT)

S6 Data Prediction results for VIPs with the undefined directions. (TXT)
