# Supplementary material for: In silico prediction of HIV-1-host molecular interactions and their directionality
Source: PLoS Comput Biol. 2022 Feb 8;18(2):e1009720. doi: 10.1371/journal.pcbi.1009720 (PMC8856524; doi:10.1371/journal.pcbi.1009720)
Supplement: S1 Appendix — (PDF) [file pcbi.1009720.s001.pdf]

S1 Appendix. Characterisation of features linked to alternative splicing and evolution

Alternative splicing allows individual genes to generate multiple messenger RNA (mRNA). It has been widely reported in an estimated 95% of multi-exon human genes [1]. This posttranscriptional process contributes to transcript variation and can produce protein isoforms with related or distinct functions, thus it has been regarded as an important driver of the evolution of phenotypic complexity [2,3]. We found that approximately 88% of our collected human genes had more than one transcript after undergoing alternative splicing. Non-VIP genes were significantly enriched with small numbers of transcripts (<6, Pearson's Chi-squared test: P=9.1E-95) or protein-coding transcripts (<4, Pearson's Chi-squared test: P=1.2E-98) while VIP genes tended to have a large number of transcripts or protein-coding transcripts (**Fig A**). We found approximately 30% of non-VIP genes were non-polymorphic (only having one protein-coding transcript or open reading frame), but for VIPs, the ratio reduced to 12%. This provided a strong signal of inhibition for HIV-1 infection in the proteins of interest (Pearson’s Chi-squared test: P=2.8E-71). On the other hand, human proteins coding from genes with high polymorphism were more likely to interact with HIV-1 (>=4, Mann–Whitney U test: P=1.1E-27) and the interacting direction tended to be ‘backward’ or ‘bidirectional’ rather than ‘forward’ if the polymorphism reached a very high level (Mann–Whitney U test: P=0.056, 0.007, respectively).

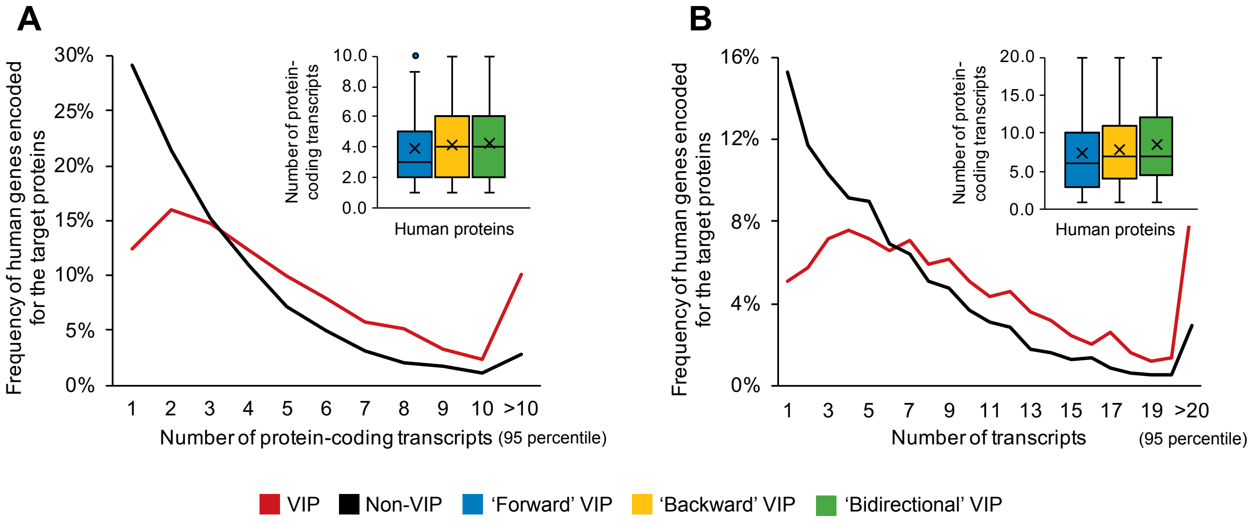

Fig A. Representation of features about alternative splicing. Insets in panels A and B represent the major distribution of expression values (from the first to the third quartile); outliers were defined by expression values higher than two-fold of the third quartile; the cross symbol marked the position of average expression value including outliers; upper and lower whiskers showed the maximum and minimum expression values excluding outliers. Abbreviations: HIV-1, human immunodeficiency virus type 1; VIP, HIV-1 interacting human protein; non-VIP, non-HIV-1 interacting human protein.

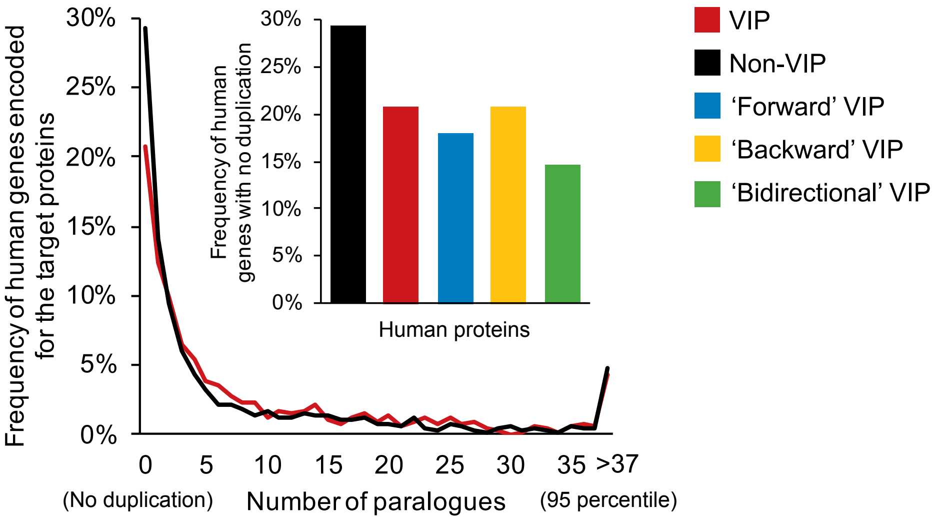

Fig B. A breakdown of paralogues for different human proteins. Abbreviations: HIV-1, human immunodeficiency virus type 1; VIP, HIV-1 interacting human protein; non-VIP, non-HIV-1 interacting human protein.

The duplication events of genes accumulate loss-of-function mutations (degeneration) within paralogues and hence promote the subfunctionalization of the ancestral genes [4,5]. The paralogue data within human species indicated that proteins encoded by singleton genes were less likely to interact with HIV-1 (Pearson's Chi-squared test: P=2.1E-16) (**Fig B**). Furthermore, human proteins produced by genes with more duplications had a higher chance to be targeted by HIV-1 (Mann–Whitney U test: P=9.7E-4). These results suggested an association between less-degenerative mutations and HIV-1 infections. The orthologue data (**Fig C**) indicated that HIV-1 molecules were more likely to interact with human proteins encoded from conserved genes (measured by dN/dS ratios, Mann–Whitney U test: P=9.5E-7, 2.8E-10, 4.9E=6, 2.9E-6, respectively). Comparing with 'forward' VIP genes, 'backward' VIPs were generally more conserved, which showed significant differences in the dN/dS ratio within human-gorilla, human-orangutan or human-gibbon orthologues (Mann–Whitney U test: P=0.038, 0.004, 0.029, respectively).

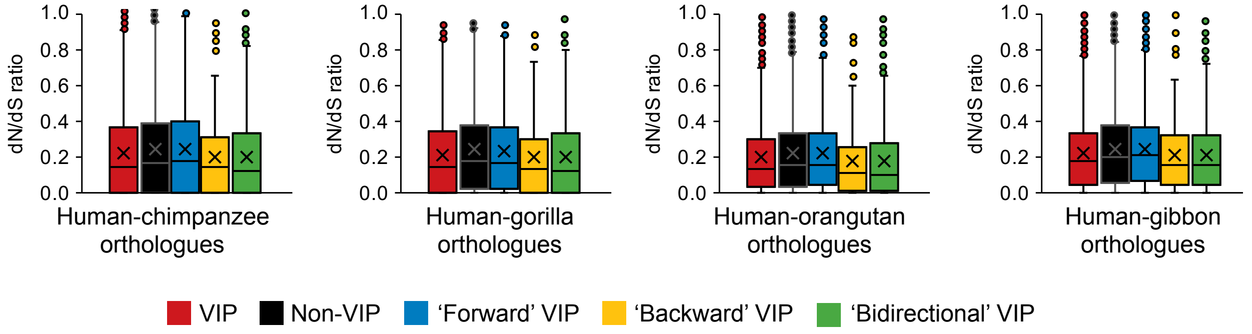

Fig C. Representation of dN/dS ratio among human and four homininae genomes. Insets here represented the major distribution of expression values (from the first to the third quartile); outliers were defined by expression values higher than two-fold of the third quartile; cross symbol marked the position of average expression value including outliers; upper and lower whiskers showed the maximum and minimum expression values excluding outliers. Abbreviations: HIV-1, human immunodeficiency virus type 1; VIP, HIV-1 interacting human protein; non-VIP, non-HIV-1 interacting human protein; dN, non-synonymous substitutions; dS, synonymous substitutions.

In summary, some evolution-related properties of human proteins influenced their propensity to interact with HIV-1. High number of protein-coding transcripts, higher duplication rates and evolutionary conservation were found to have a positive influence on the human proteins, promoting HIV-1-host PPIs.

References

1. Pan Q, Shai O, Lee LJ, Frey BJ, Blencowe BJ. Deep surveying of alternative splicing complexity in the human transcriptome by high-throughput sequencing. Nat Genet. 2008; 40(12): 1413-1415. https://doi.org/10.1038/ng.259 PMID: 18978789

2. Wang ET, Sandberg R, Luo S, Khrebtukova I, Zhang L, Mayr C, et al. Alternative isoform regulation in human tissue transcriptomes. Nature. 2008; 456(7221): 470-476. https://doi.org/10.1038/nature07509 PMID: 18978772

3. Keren H, Lev-Maor G, Ast G. Alternative splicing and evolution: diversification, exon definition and function. Nature Reviews Genetics. 2010; 11(5): 345-355. https://doi.org/10.1038/nrg2776 PMID: 20376054

4. Hittinger CT, Carroll SB. Gene duplication and the adaptive evolution of a classic genetic switch. Nature. 2007; 449(7163): 677-681. https://doi.org/10.1038/nature06151 PMID: 17928853

5. Freilich S, Massingham T, Blanc E, Goldovsky L, Thornton JM. Relating tissue specialization to the differentiation of expression of singleton and duplicate mouse proteins. Genome Biol. 2006; 7(10): 1-14. https://doi.org/10.1186/gb-2006-7-10-r89 PMID: 17029626
