# Supplementary material for: In silico prediction of HIV-1-host molecular interactions and their directionality
Source: PLoS Comput Biol. 2022 Feb 8;18(2):e1009720. doi: 10.1371/journal.pcbi.1009720 (PMC8856524; doi:10.1371/journal.pcbi.1009720)
Supplement: S2 Appendix — (PDF) [file pcbi.1009720.s002.pdf]

S2 Appendix. Characterisation of features in different sequence patterns

From the collected data, we found obvious enrichment of adenine in VIP genes (Mann–Whitney U test: P=1.9E-20) (**Fig D**). The majority of adenine-related nucleobase groups are enriched in coding sequences (CDS) of VIP genes (**Fig D**, **Fig EA**). Alternatively, cytosine tended to be depleted in VIP genes (Mann–Whitney U test: P=1.3E-10) (**Fig D**) thus three cytosine-starting nucleobases groups, i.e., CpT, CpC and CpG, all showed significant depletion in the CDS of VIP genes (**Fig D**, **Fig EA**). Although thymine was slightly depleted in the CDS of human genes (**Fig D**), it still made an important contribution in classifying VIPs and non-VIPs from the perspective of codon usage (**Fig EC**). Among the 28 VIP-preferred codons, 20 codons contained at least one thymine.

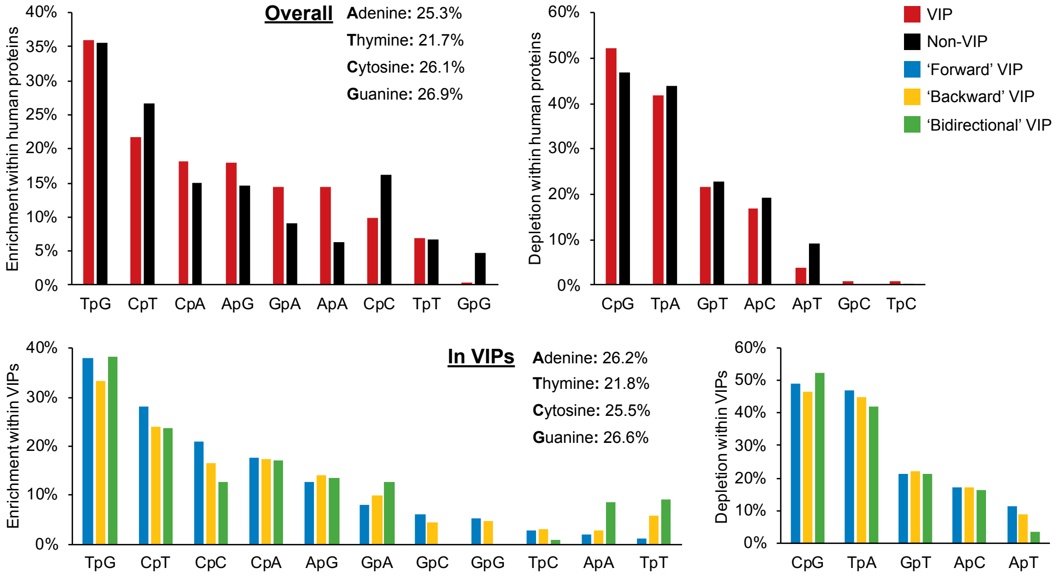

Fig D. Enrichment and depletion of nucleotides linked by phosphodiester bonds in the group of human proteins or VIPs. Abbreviations: HIV-1, human immunodeficiency virus type 1; VIPs, HIV-1 interacting human proteins; non-VIPs, non-HIV-1 interacting human proteins.

Enrichment of adenine, depletion of cytosine, and differential codon usage preferences of VIP genes all influenced the distribution of amino acids in the protein sequence [1], which also contributed to the signal distinguishing VIPs from non-VIPs. As shown in **Fig EB**, we found acidic or negatively charged amino acid: aspartic acid (D) and glutamic acid (E), amide amino acid: asparagine (N) and glutamine (Q) were all significantly enriched in VIPs. Hydrophilicity, polarity, or even the size of amino acids are presumably good features to identify VIPs (**S3 Data**). Differences between 'backward' and 'forward' VIPs were generally not obvious from the perspective of nucleotide compositions, codon usages, or amino acid compositions. However, differences between 'bidirectional' and 'forward' VIPs

were notable in 56% of nucleotide composition features, 41% of codon usage features, and 51% of amino acid composition features.

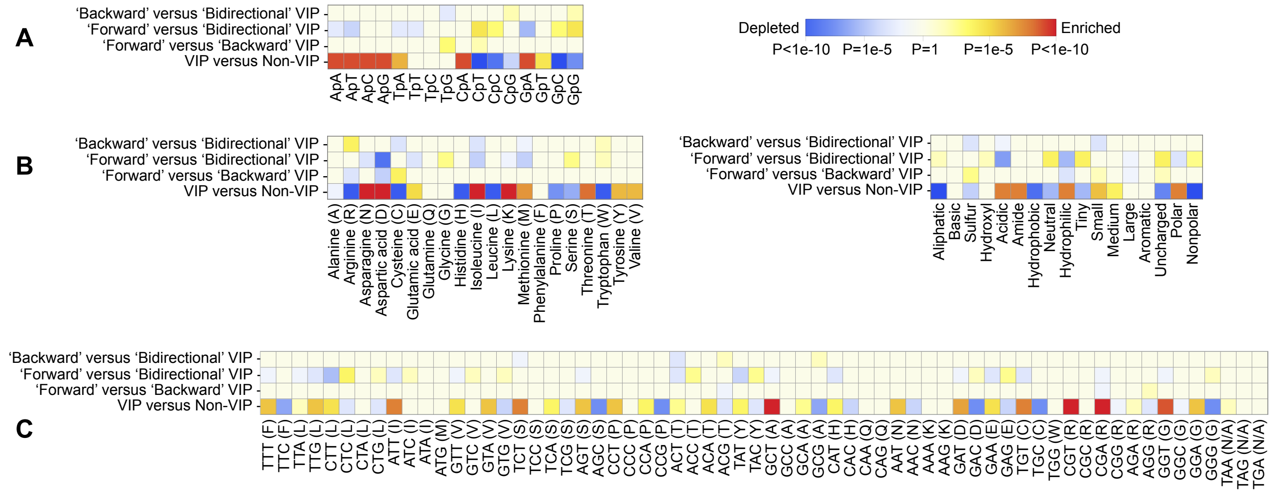

Fig E. The difference of (A) nucleobase groups linked with phosphodiester bonds, (B) amino acid compositions and (C) codon usages in different classes. Detailed data about the heat maps are provided in **S3 Data**. Abbreviations: HIV-1, human immunodeficiency virus type 1; VIP, HIV-1 interacting human protein; non-VIPs, non-HIV-1 interacting human protein.

We found that some sequence patterns were highly similar to each other, which might result from small deletions, insertions or mutations [2]. Therefore, we integrated these sequence patterns and obtained 206 SLiMs enriched in VIPs and 'backward' VIPs. Some top-ranked SLiMs are listed in **Table A**. The dash symbol in a SLiM represented a position occupied by no or one random amino acid. The results showed that there were many specific SLiMs presenting in VIPs, but they were scarce in non-VIPs. Some elements in the SLiM were conserved and less influenced by random amino acid changes, which might be related to a contributing signal from their evolutionary history [3]. Alanine (A) was found at high frequency (74%) in VIP-SLiMs, followed by lysine (K) and glutamic acid (E) observed in 60% and 55% of VIP-enriched SLiMs. Both K and E were significantly enriched in VIPs (**Fig EB**), but aspartic acid (D) and isoleucine (I) seemed to be irrelevant to the conserved region even if they were highly enriched in VIP sequences (**S3 Data**). Differences in SLiMs were also observed between 'backward' and 'forward' VIPs. Amino acid A and leucine (L) seemed to be important to 'backward' VIPs as they were found in 51% and 55% of the enriched SLiMs (**S3 Data**). Additionally, we found notable differences in the overall abundance of SLiMs between the compared classes. We found 54 VIP-enriched SLiMs in the sequence of a VIP, namely plectin (PLEC) but the highest co-occurrence frequency of these SLiMs only reached 42 within the group of non-VIPs (found in the spen

family transcriptional repressor, SPEN). Around 90% of VIPs contained at least one VIP-enriched SLiM versus 82% of non-VIPs. The difference of cooccurrence status was also observed in 'backward' VIP-enriched SLiMs. The cumulative frequency of 'backward' VIP-enriched SLiMs was 97.9% in 'backward' VIPs and reduced to 92.5% in 'forward' VIPs (**Fig F**).

Table A. Top 20 enriched SLiMs in VIPs and backward VIPs.

SLiM	VIP/ non-VIP	P-value[a]	Expression[b]	SLiM	'Backward'/ 'Forward' VIP	P-value	Expression
AK-K-E	200/257	9.4E-14	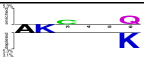	P-E-R-V	25/37	4.7E-08	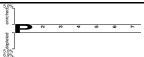
AK-A-E	201/266	7.0E-13	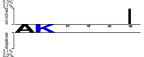	L-D-T-R	27/50	1.5E-06	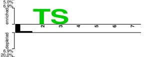
E-AK-K	220/310	6.4E-12	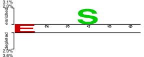	L-R-I-G	20/33	6.8E-06	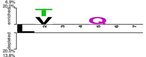
EKE-K	221/315	1.3E-11	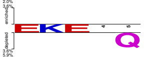	P-D-SS	25/50	1.5E-05	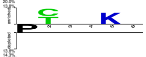
EK-A-K	238/351	2.8E-11	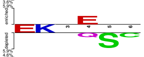	D-K-Q-E	19/32	1.6E-05	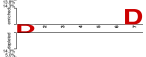
AA-K-K	184/249	3.1E-11	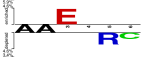	S-L-IS	22/41	1.7E-05	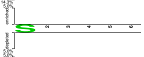
D-Q-L-K	217/312	3.9E-11	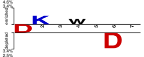	G-SAA	21/39	2.6E-05	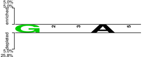
D-L-K-D	241/359	4.5E-11	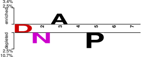	RS-G-S	21/39	2.6E-05	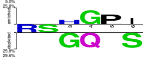
A-D-D-E	204/288	4.6E-11	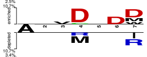	R-RS-L	23/46	3.5E-05	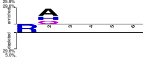
K-K-E-P	240/359	6.9E-11	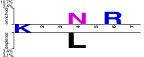	DFF	20/37	3.9E-05	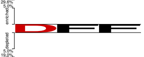
G-K-K-V	236/352	8.1E-11	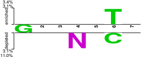	F-H-M	20/37	3.9E-05	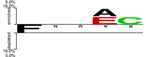
K-G-K-G	239/358	8.4E-11	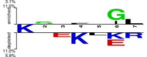	T-SL-T	21/41	5.6E-05	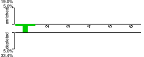
E-MN	238/357	1.0E-10	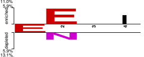	KV-A-E	20/38	5.8E-05	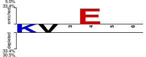
E-DL-K	240/363	1.6E-10	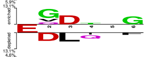	LG-I-S	21/42	8.1E-05	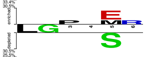
A-K-V-K	217/320	2.3E-10	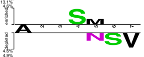	R-S-G-P	21/42	8.1E-05	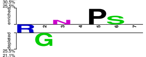
T-E-E-T	235/356	2.8E-10	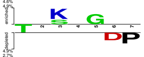	G-LS-K	19/36	8.7E-05	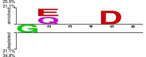
GD-M	235/357	3.5E-10	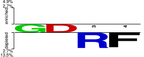	Q-K-P-L	22/46	1.1E-04	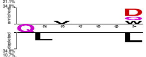
G-K-K-G	229/346	4.1E-10	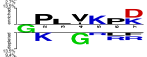	L-Y-L-E	23/50	1.3E-04	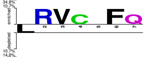
G-G-T-T	236/360	4.3E-10	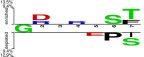	N-L-R-S	22/47	1.5E-04	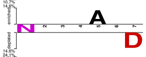
D-E-V-K	216/321	4.4E-10	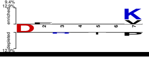	Q-S-S-K	21/44	1.6E-04	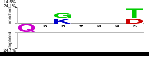

[a]*expression differences of SLiMs were assessed through the Pearson's Chi-squared tests on different classes;* [b]*expression of amino acids in the sequence segments containing a target SLiM; for VIP-enriched SLiMs, the positive and negative segment sets were extracted from VIPs and non-VIPs, respectively; for 'backward'-enriched SLiMs, the positive and negative segment sets were extracted from 'backward' and 'forward' VIPs, respectively.* Abbreviations: HIV-1, human immunodeficiency virus type 1; VIP, HIV-1 interacting human protein; non-VIP, non-HIV-1 interacting human protein; SLiM, protein short linear motif.

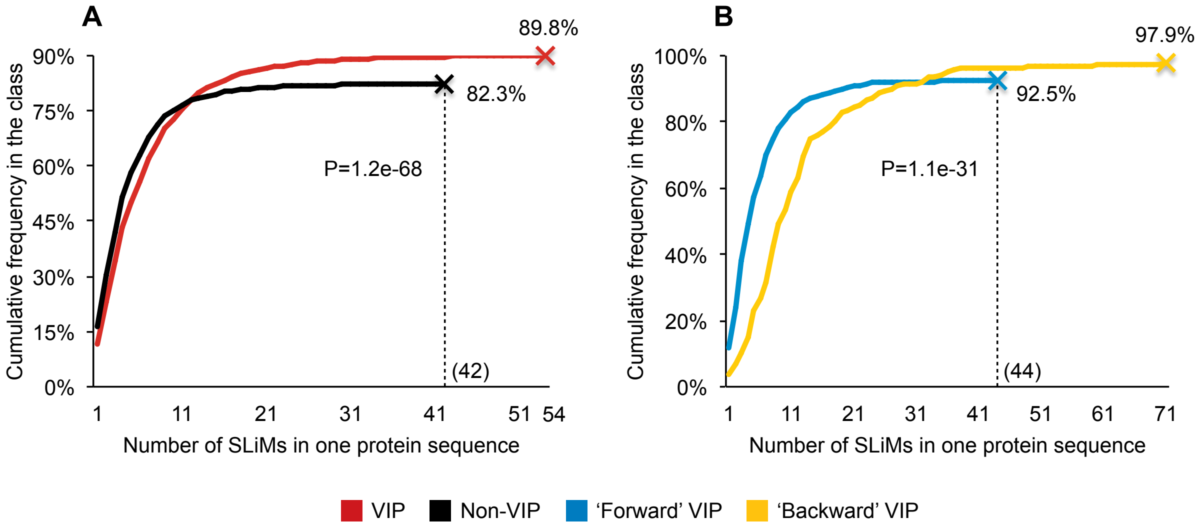

Fig F. Expression of (A) 85 VIP-enriched SLiMs in human proteins and (B) 121 backward VIP-enriched SLiMs in the forward and backward VIPs. Difference between these expressions are evaluated with the Mann–Whitney U tests. Abbreviations: HIV-1, human immunodeficiency virus type 1; VIPs, HIV-1 interacting human proteins; non-VIPs, non-HIV-1 interacting human proteins; SLiMs, protein short linear motifs.

Intrinsically disordered regions have a broad occurrence in proteins, allowing the same polypeptide to be involved in different PPIs [4]. Characterised by their biased amino acid composition and low sequence complexity, intrinsically disordered proteins lack the ability to fold spontaneously into stable secondary and well-packed tertiary structures. However, they still play an important role in many biological activities. Based on the result given by the IUPred [5], 92% of VIPs contained at least one disorder region while 89% of non-VIPs were disordered (Pearson's Chi-squared test: P=5.3E-5). Distributions of disorder regions were not distinguishable when comparing VIPs with non-VIPs (**Fig G**) but were slightly different in VIPs with distinct directionality (**Fig H**). We found that 'backward' VIPs were less likely to form disorder regions close to the beginning or end of their sequences. Disorder regions were less frequent in the middle of 'bidirectional' VIP sequences and showed great depletion at the end of the VIP sequence (**Fig HA**). The representation of some amino acids, e.g., serine (S), threonine (T) (**Fig HB**), E, and K (**Fig HD**), were biased by the directionality of HIV-1-host molecular interactions. We assumed the results of the Espritz [6] might be more useful since they could link the information of VIP-enriched SLiMs and disorder expression in the 'backward' VIPs. As mentioned in the manuscript, amino acids K and E are important to the pattern of VIP-enriched

SLiMs (**S3 Data**), and they had a higher chance to be found in disordered regions in the sequence of 'backward' VIPs.

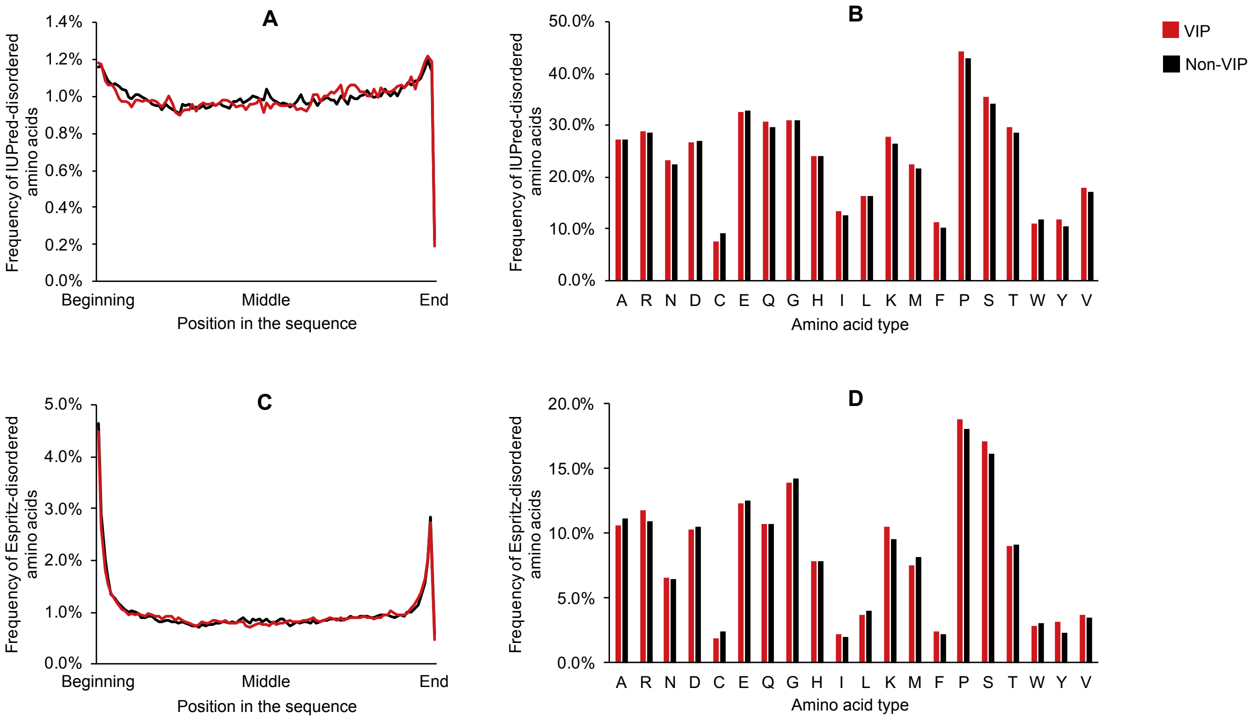

Fig G. Disorder status in different human proteins. (A) and (B) show the results calculated by IUPred [5] while (C) and (D) show the results calculated by Espritz [6]. Abbreviations: HIV-1, human immunodeficiency virus type 1; VIPs, HIV-1 interacting human proteins; non-VIPs, non-HIV-1 interacting human proteins.

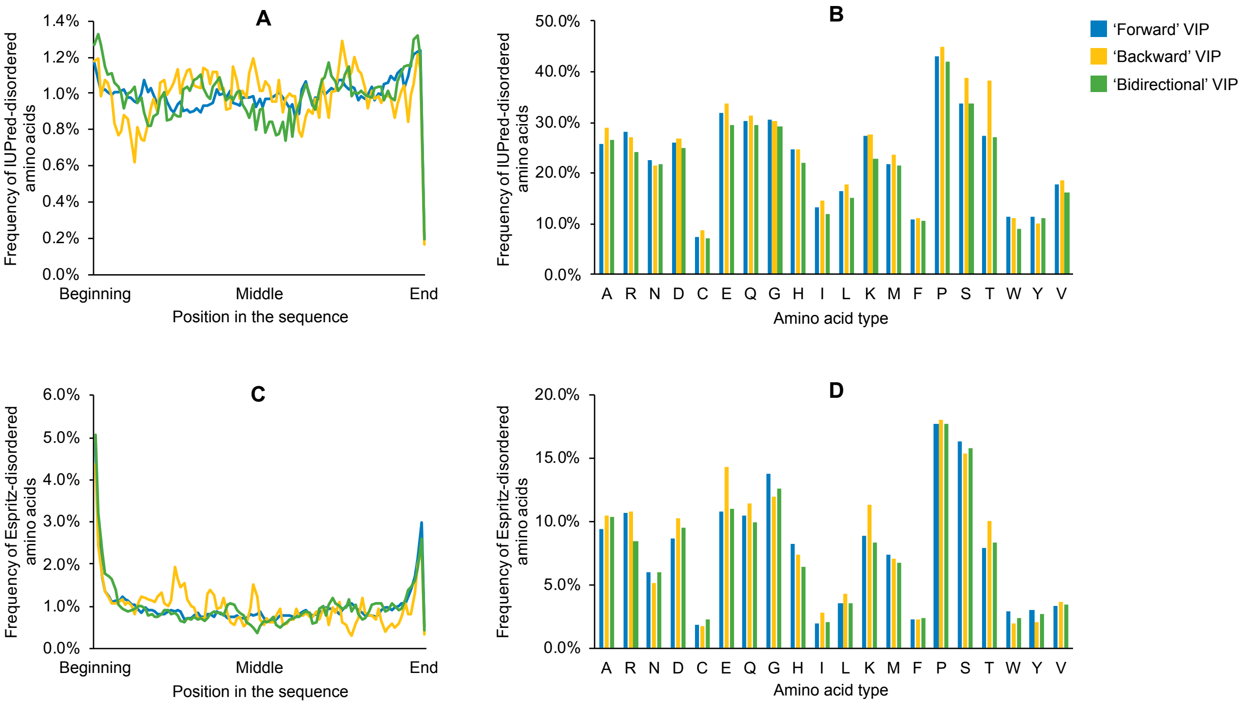

Fig H. Disorder status in different VIPs. (A) and (B) show the results calculated by IUPred [5] while (C) and (D) show the results calculated by Espritz [6]. Abbreviations: HIV-1, human immunodeficiency virus type 1; VIPs, HIV-1 interacting human proteins.

Briefly, VIPs and non-VIPs showed some significant differences in their sequence patterns from the nucleobase composition to SLiMs. It gave an acceptable answer to explain the reason why some human host proteins could interact with multiple HIV-1 proteins. Meanwhile, pro-viral and pro-host signs of VIPs were also reflected by special sequence patterns and intrinsic disorder status in the protein sequence.

References

1. Pearson WR. Finding protein and nucleotide similarities with FASTA. Curr Protoc Bioinformatics. 2016; 53(1): 3-9. https://doi.org/10.1002/0471250953.bi0309s04 PMID: 18428723

2. Vens C, Rosso M-N, Danchin EG. Identifying discriminative classification-based motifs in biological sequences. Bioinformatics. 2011; 27(9): 1231-1238. https://doi.org/10.1093/bioinformatics/btr110 PMID: 21372086

3. Davey NE, Cyert MS, Moses AM. Short linear motifs–ex nihilo evolution of protein regulation. Cell Commun Signal. 2015; 13(1): 1-15. https://doi.org/10.1186/s12964-015-0120-z PMID: 26589632

4. Wright PE, Dyson HJ. Intrinsically disordered proteins in cellular signalling and regulation. Nat Rev Mol Cell Biol. 2015; 16(1): 18-29. https://doi.org/10.1038/nrm3920 PMID: 25531225

5. Mészáros B, Erdős G, Dosztányi Z. IUPred2A: context-dependent prediction of protein disorder as a function of redox state and protein binding. Nucleic Acids Res. 2018; 46(W1): W329-W337. https://doi.org/10.1093/nar/gky384 PMID: 29860432

6. Walsh I, Martin AJ, Di Domenico T, Tosatto SC. ESpritz: accurate and fast prediction of protein disorder. Bioinformatics. 2012; 28(4): 503-509. https://doi.org/10.1093/bioinformatics/btr682 PMID: 22190692
