# Supplementary material for: In silico prediction of HIV-1-host molecular interactions and their directionality
Source: PLoS Comput Biol. 2022 Feb 8;18(2):e1009720. doi: 10.1371/journal.pcbi.1009720 (PMC8856524; doi:10.1371/journal.pcbi.1009720)
Supplement: S3 Appendix — (PDF) [file pcbi.1009720.s003.pdf]

S3 Appendix. Characterisation of features in annotation and network profiles

The Gene Ontology is a resource of knowledge unifying the representation of gene and gene product attributes. It is reported to be one of the strongest indicators of interacting proteins [1]. Compared with the annotation profile of non-VIPs, we found some GO terms, e.g., cytokine-mediated signalling pathway (GO:0019221), protein binding (GO:0005515), and nucleoplasm (GO:0005654) were highly enriched in the GO profile of VIPs. In order to reduce the dependency of our knowledge models on the annotated GO profile, we mapped the collected GO terms through the derivation tree and cataloged them into 66 domains representing child terms of biological process, molecular function and cellular component (**S3 Data**). Based on these 'new' GO profiles, we found an estimated 90% of VIPs were involved in the cellular process while the ratio reduced to two-thirds in non-VIPs (Pearson's Chi-squared test: P=1.9E-123) (**Fig I**). The difference of binding activities was also observed between VIPs and non-VIPs (P=1.9E-84) (**Fig J**). This is not surprising since the majority of VIPs were placed in key positions with a high degree or betweenness centrality within the human interactome (**S3 Data**). Additionally, we also found some clues in the catalogued GO profiles, which might help for the classification of VIPs with different directionality. For instance, approximately 77% of 'bidirectional' VIPs could raise a response to stimulus but the percentage for 'forward' VIPs, 'backward' VIPs, and non-VIPs only reached 47%, 29% and 20%, respectively (**Fig I**, **S3 Data**). 'Backward' or 'bidirectional' VIPs were more likely to be found in organelles as opposed to 'forward' VIPs and non-VIPs (P=7.3E-5, 1.6E-7, 2.0E-70, respectively) (**Fig K**, **S3 Data**).

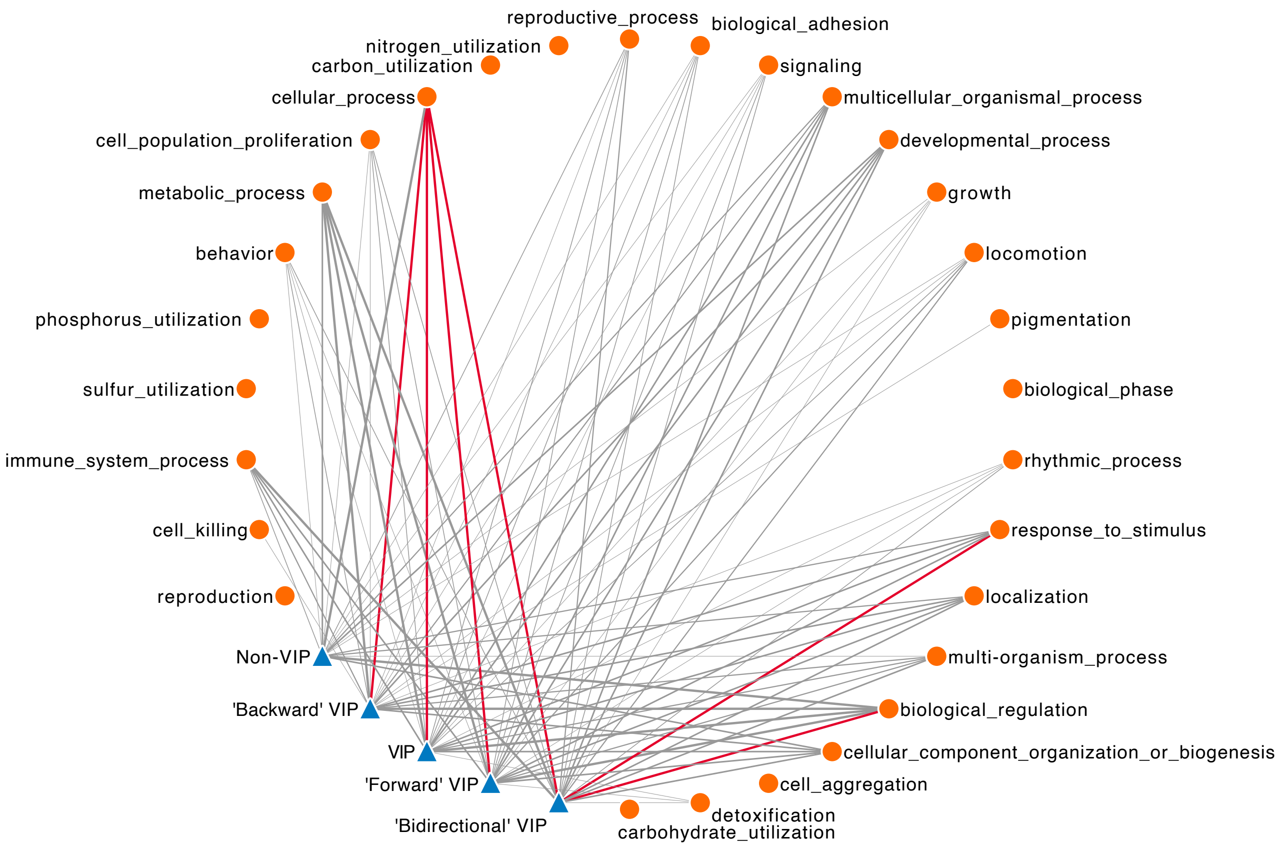

Fig I. GO annotation status of different VIPs under the catalogue of biological process. Bolder lines indicate stronger relationship between human proteins and GO child terms. Red lines indicate a GO child term is related to more than 75% of human proteins. Detailed data about the GO involvement in different human proteins are provided in **S3 Data**. Abbreviations: HIV-1, human immunodeficiency virus type 1; VIP, HIV-1 interacting human protein; GO, gene ontology.

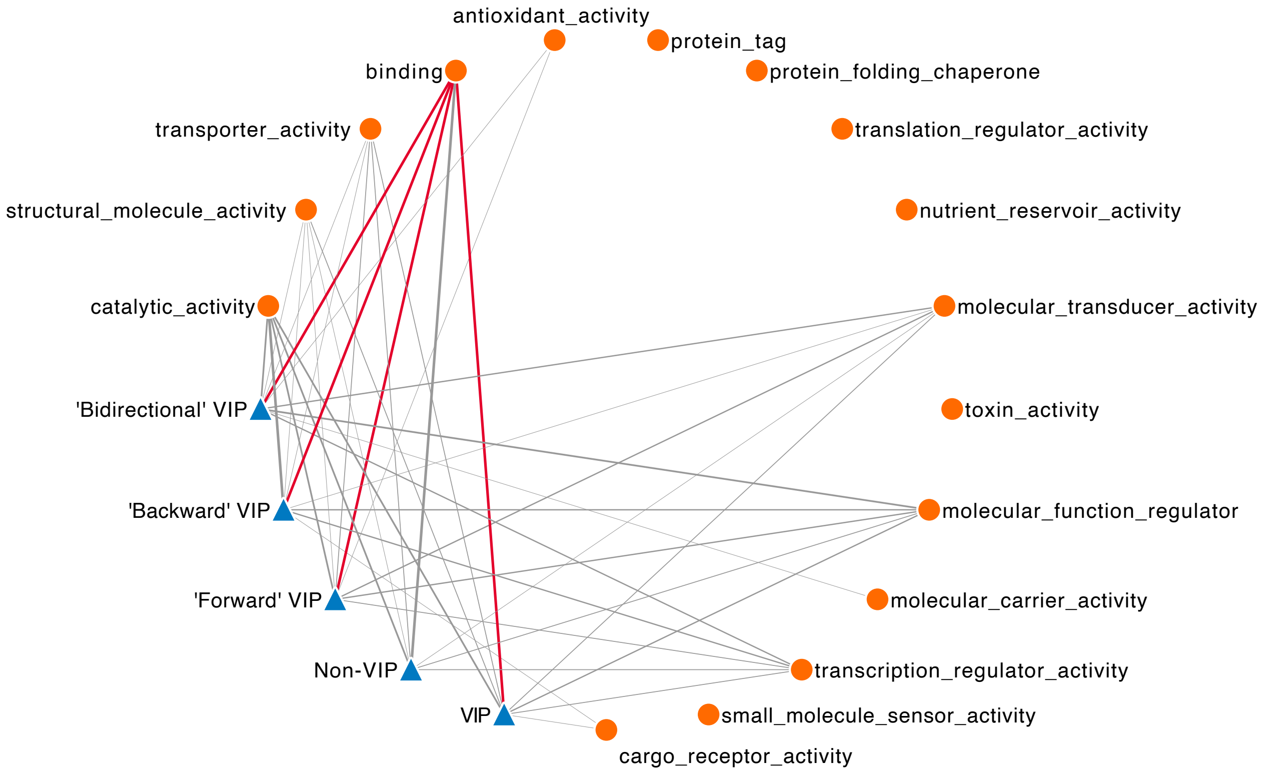

Fig J. GO annotation status of different VIPs under the catalogue of molecular function. Bolder lines indicate stronger relationship between human proteins and GO child terms. Red lines indicate a GO child term is related to more than 75% of human proteins. Detailed data about the GO involvement in different human proteins are provided in **S3 Data**. Abbreviations: HIV-1, human immunodeficiency virus type 1; VIP, HIV-1 interacting human protein; GO, gene ontology.

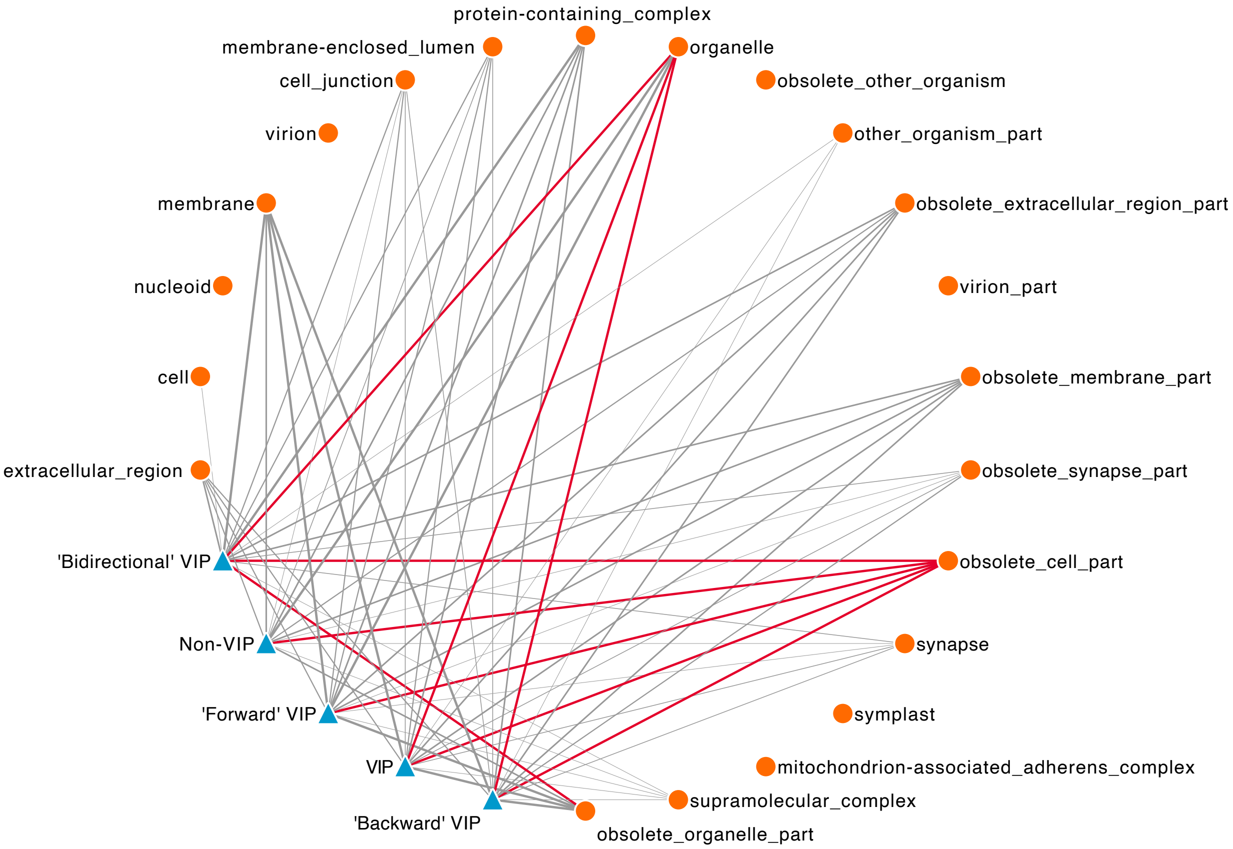

Fig K. GO annotation status of different VIPs under the catalogue of cellular component. Bolder lines indicate stronger relationship between human proteins and GO child terms. Red lines indicate a GO child term is related to more than 80% of human proteins. Detailed data about the GO involvement in different human proteins are provided in **S3 Data**. Abbreviations: HIV-1, human immunodeficiency virus type 1; VIP, HIV-1 interacting human protein; GO, gene ontology.

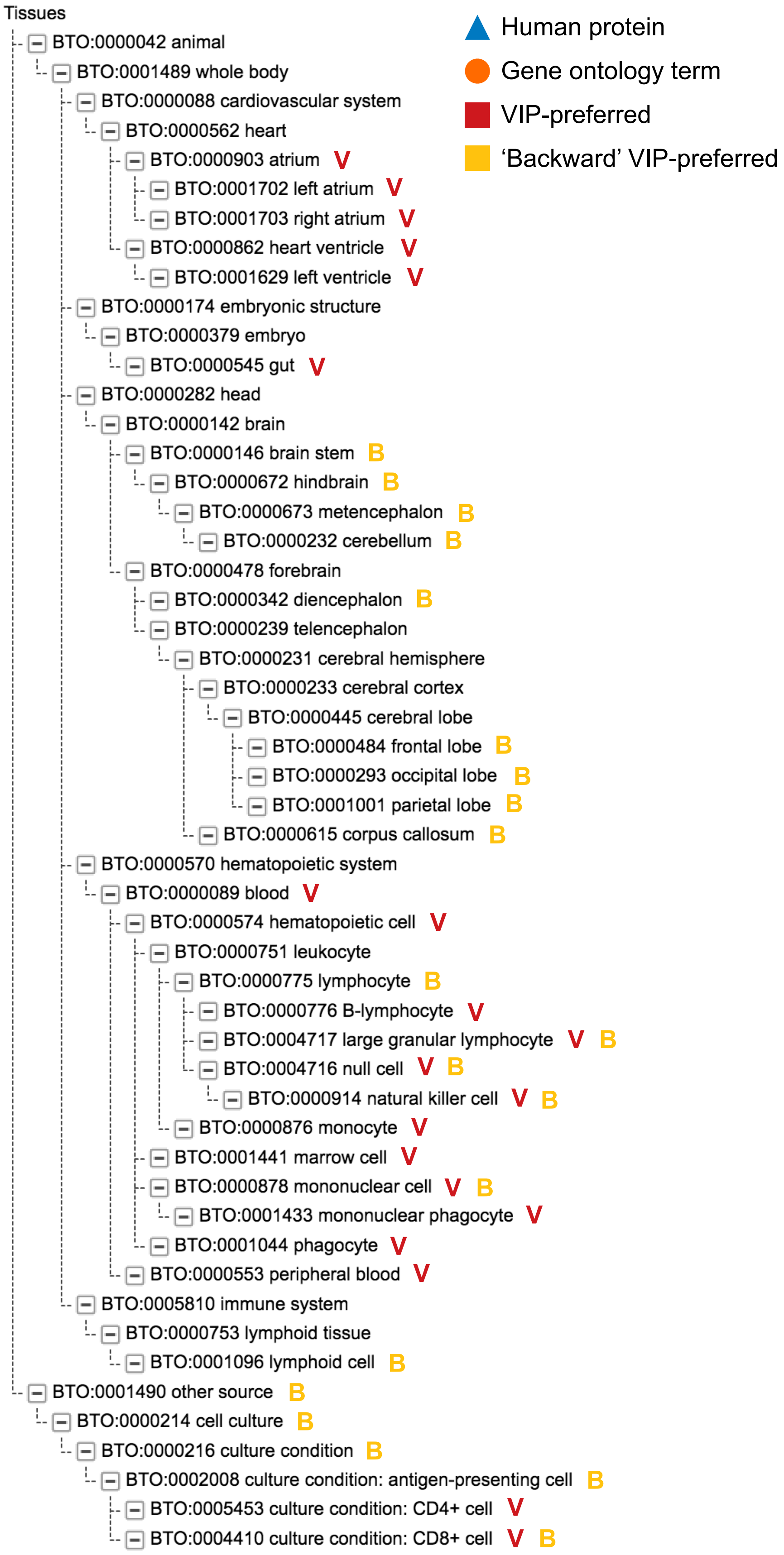
Tissues
BTO:0000042 animal
BTO:0001489 whole body
BTO:0000088 cardiovascular system
BTO:0000562 heart
BTO:0000903 atrium V
BTO:0001702 left atrium V
BTO:0001703 right atrium V
BTO:0000862 heart ventricle V
BTO:0001629 left ventricle V
BTO:0000174 embryonic structure
BTO:0000379 embryo
BTO:0000545 gut V
BTO:0000282 head
BTO:0000142 brain
BTO:0000146 brain stem B
BTO:0000672 hindbrain B
BTO:0000673 metencephalon B
BTO:0000232 cerebellum B
BTO:0000478 forebrain
BTO:0000342 diencephalon B
BTO:0000239 telencephalon
BTO:0000231 cerebral hemisphere
BTO:0000233 cerebral cortex
BTO:0000445 cerebral lobe
BTO:0000484 frontal lobe B
BTO:0000293 occipital lobe B
BTO:0001001 parietal lobe B
BTO:0000615 corpus callosum B
BTO:0000570 hematopoietic system
BTO:0000089 blood V
BTO:0000574 hematopoietic cell V
BTO:0000751 leukocyte
BTO:0000775 lymphocyte B
BTO:0000776 B-lymphocyte V
BTO:0004717 large granular lymphocyte V B
BTO:0004716 null cell V B
BTO:0000914 natural killer cell V B
BTO:0000876 monocyte V
BTO:0001441 marrow cell V
BTO:0000878 mononuclear cell V B
BTO:0001433 mononuclear phagocyte V
BTO:0001044 phagocyte V
BTO:0000553 peripheral blood V
BTO:0005810 immune system
BTO:0000753 lymphoid tissue
BTO:0001096 lymphoid cell B
BTO:0001490 other source B
BTO:0000214 cell culture B
BTO:0000216 culture condition B
BTO:0002008 culture condition: antigen-presenting cell B
BTO:0005453 culture condition: CD4+ cell V
BTO:0004410 culture condition: CD8+ cell V B
Human protein
Gene ontology term
VIP-preferred
'Backward' VIP-preferred

Fig L. Top 20 tissues preferred by VIPs and 'backward' VIPs. Detailed data about the top 20 tissue tropisms are provided in **S3 Data**. Abbreviations: HIV-1, human immunodeficiency virus type 1; VIP, HIV-1 interacting human protein.

From the perspective of tissue tropisms, VIPs preferred heart- or hematopoietic system-related tissue. The most significant difference between VIP and non-VIP was found in their association with monocyte (nongranular phagocytic leukocyte) where relatively long-lived macrophages were derived (**Fig L**, **S3 Data**) (P=1.7E-137) [2]. The same differences were found in phagocyte that engulfs foreign material [3] and immature blood cells that develop in the bone marrow [4]. In the tissue group of antigen-presenting cells, CD4+ and CD8+ cells were favoured by VIPs, which showed a strong relationship between a virus invading and the immune responses (P=5.1E-131 and 5.8E-114, respectively) [5,6]. Compared with 'forward' VIPs, 'backward' VIPs were less involved in the hematopoietic system, but they were more expressed in brain-related tissues, such as the brain stem and cerebral lobe (**S3 Data**). Cells originating from stem cells and differentiating in lymphoid tissues were favoured by 'backward' VIPs. The relationship between 'backward' VIPs and CD8+ cells were even more obvious, showing a clear relationship linking the virus invading and the host antiviral immune responses [7].

In closing, annotation profiles represented by the GO terms and tissue tropisms differentiated the biological environment between VIPs and non-VIPs. Some preferences of VIPs such as more involvement in cellular binding activities were also reflected in the human interactome.

References

1. Maetschke SR, Simonsen M, Davis MJ, Ragan MA. Gene Ontology-driven inference of protein–protein interactions using inducers. Bioinformatics. 2012; 28(1): 69-75. https://doi.org/10.1093/bioinformatics/btr610 PMID: 22057159
2. Wang X, Ye L, Hou W, Zhou Y, Wang Y-J, Metzger DS, et al. Cellular microRNA expression correlates with susceptibility of monocytes/macrophages to HIV-1 infection. Blood. 2009; 113(3): 671-674. https://doi.org/10.1182/blood-2008-09-175000 PMID: 19015395
3. Persidsky Y, Gendelman HE. Mononuclear phagocyte immunity and the neuropathogenesis of HIV-1 infection. J Leukoc Biol. 2003; 74(5): 691-701. https://doi.org/10.1189/jlb.0503205 PMID: 14595004

4. Alexaki A, Wigdahl B. HIV-1 infection of bone marrow hematopoietic progenitor cells and their role in trafficking and viral dissemination. PLoS Pathog. 2008; 4(12): e1000215. https://doi.org/10.1371/journal.ppat.1000215 PMID: 19112504

5. Caby F. CD4+/CD8+ ratio restoration in long-term treated HIV-1-infected individuals. AIDS. 2017; 31(12): 1685-1695. https://doi.org/10.1097/QAD.0000000000001533 PMID: 28700392

6. Boettler T, Spangenberg HC, Neumann-Haefelin C, Panther E, Urbani S, Ferrari C, et al. T cells with a CD4+ CD25+ regulatory phenotype suppress in vitro proliferation of virus-specific CD8+ T cells during chronic hepatitis C virus infection. J Virol. 2005; 79(12): 7860-7867. https://doi.org/10.1128/JVI.79.12.7860-7867.2005 PMID: 15919940

7. Puntel M, Barrett R, Sanderson NS, Kroeger KM, Bondale N, Wibowo M, et al. Identification and visualization of CD8+ T cell mediated IFN-γ signaling in target cells during an antiviral immune response in the brain. PLoS One. 2011; 6(8): e23523. https://doi.org/10.1371/journal.pone.0023523 PMID: 21897844
