# Supplementary figures and images for: In silico prediction of HIV-1-host molecular interactions and their directionality
Source: PLoS Comput Biol. 2022 Feb 8;18(2):e1009720. doi: 10.1371/journal.pcbi.1009720 (PMC8856524; doi:10.1371/journal.pcbi.1009720)

Number of HIV-1-host interactions per human protein

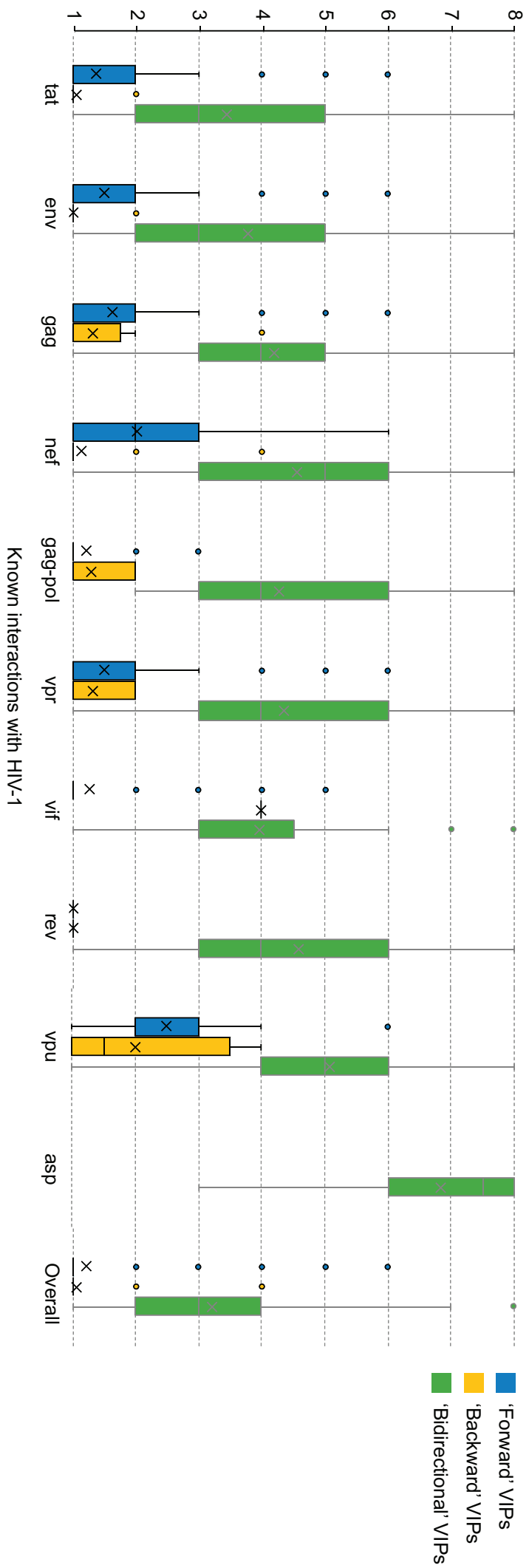

Supplement: S1 Fig — Boxes in the plot represent the major distribution of values (from the first to the third quartile); outliers were added for values higher than two-fold of the third quartile; the cross symbol marks the position of the average value including the outliers; upper and lower whiskers showed the maximum and minimum values excluding the outliers. Abbreviations: HIV-1, human immunodeficiency virus type 1; VIP, HIV-1 interacting human protein. (PDF) [file pcbi.1009720.s004.pdf]

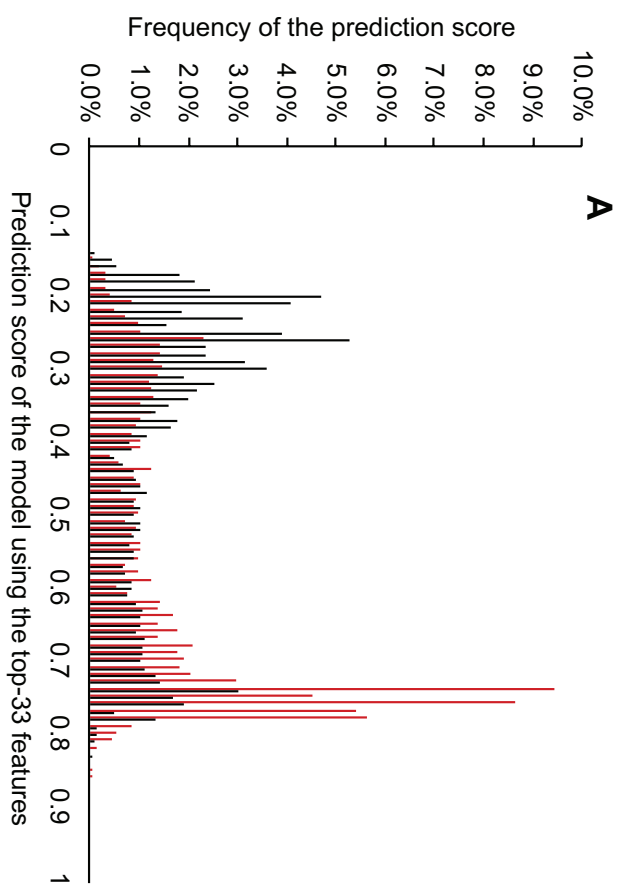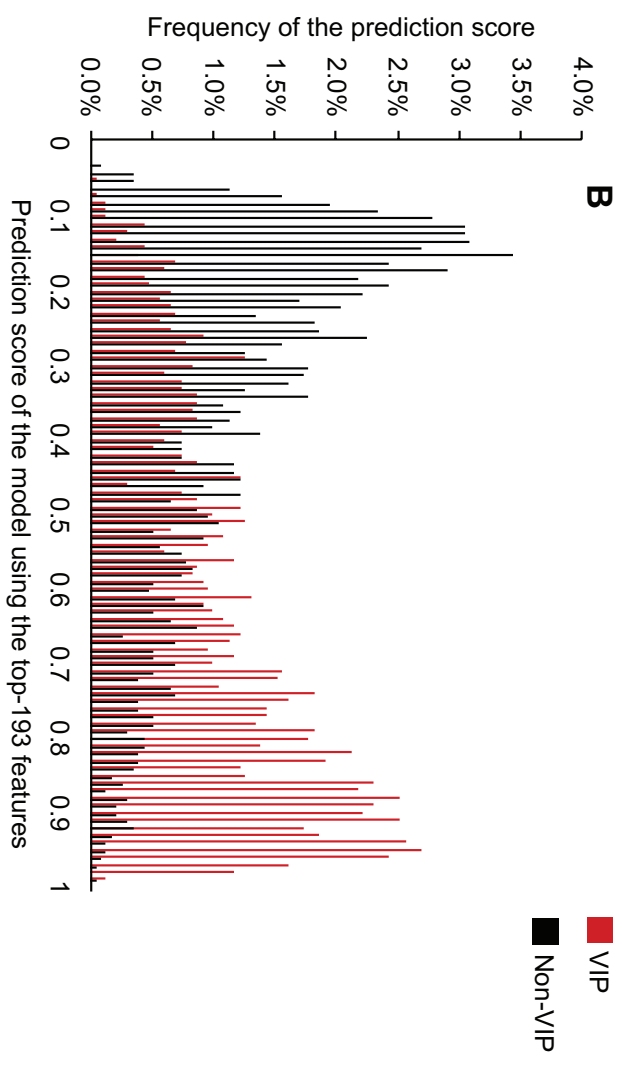

Supplement: S2 Fig — Prediction score generated by models using (A) the top-33 and (B) top-193 features on dataset S1’ over five-cross validation. Abbreviations: HIV-1, human immunodeficiency virus type 1; VIPs, HIV-1 interacting human proteins; non-VIPs, non-HIV-1 interacting human proteins. (PDF) [file pcbi.1009720.s005.pdf]

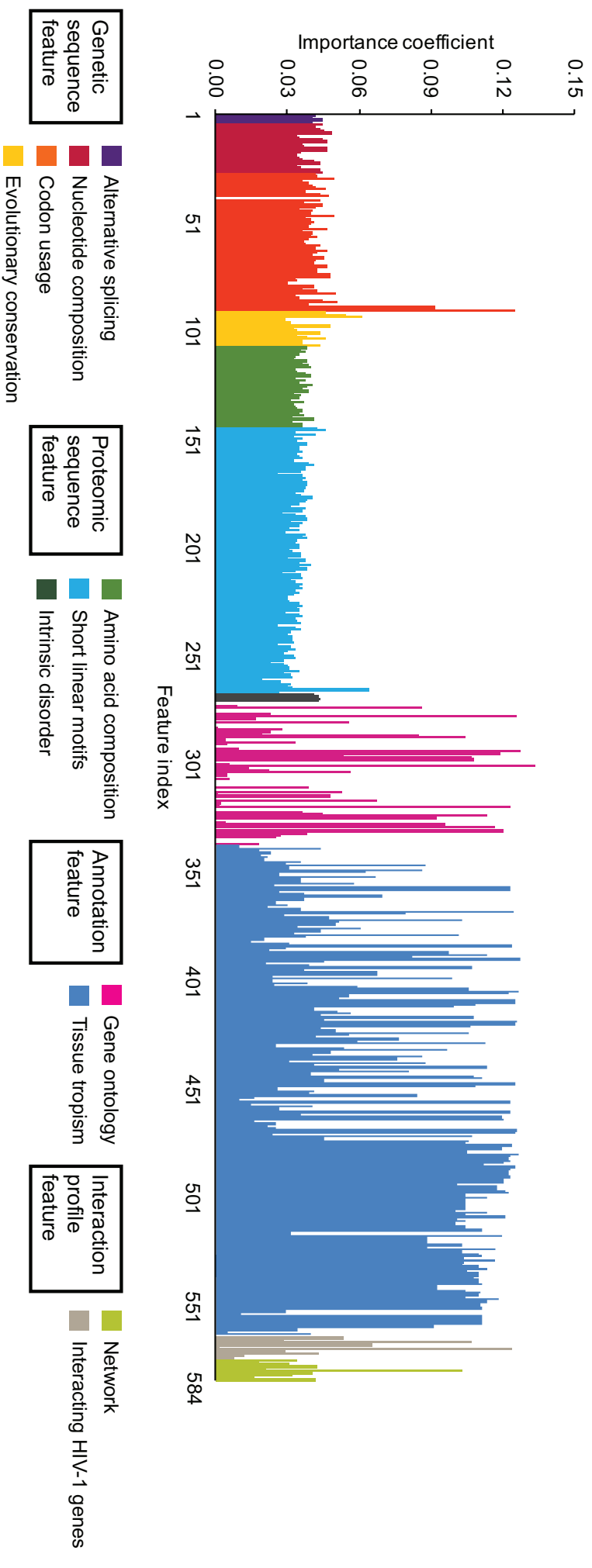

Supplement: S3 Fig — Abbreviations: The importance score of individual features is recorded by averaging the results on the balanced training datasets generated by ten-round undersampling procedures on dataset S2. HIV-1, human immunodeficiency virus type 1; VIPs, HIV-1 interacting human proteins. (PDF) [file pcbi.1009720.s006.pdf]

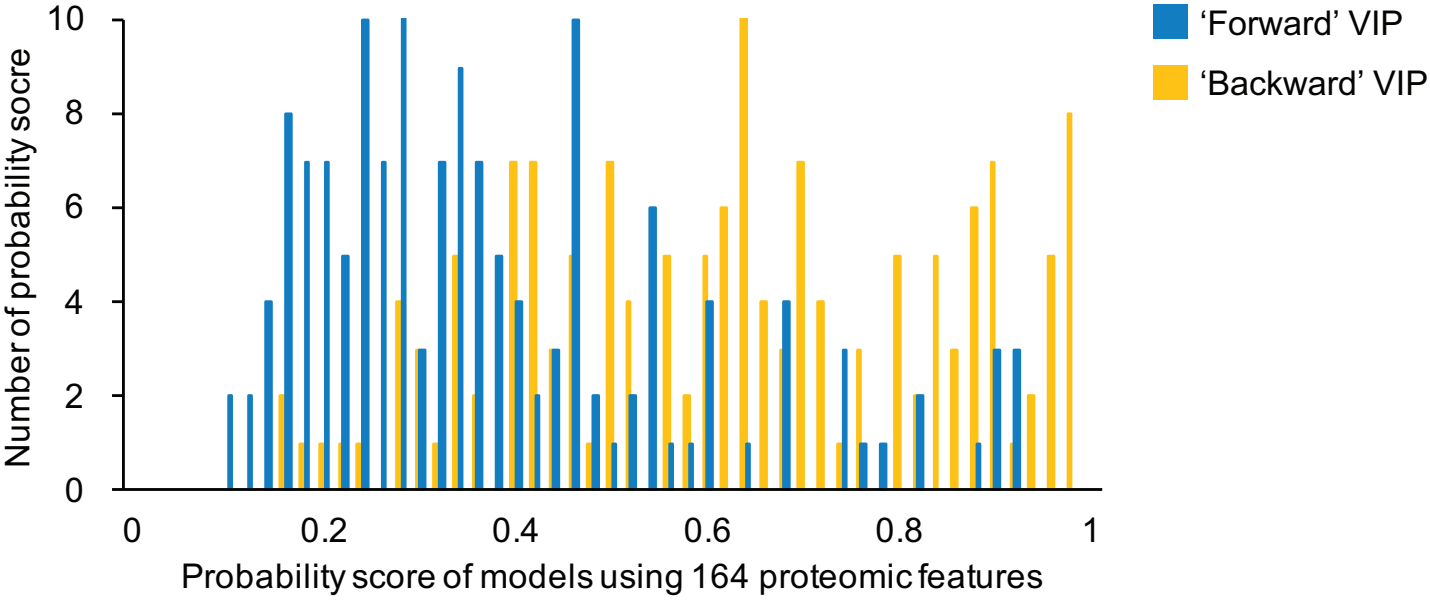

Supplement: S4 Fig — Abbreviations: HIV-1, human immunodeficiency virus type 1; VIPs, HIV-1 interacting human proteins. (PDF) [file pcbi.1009720.s007.pdf]

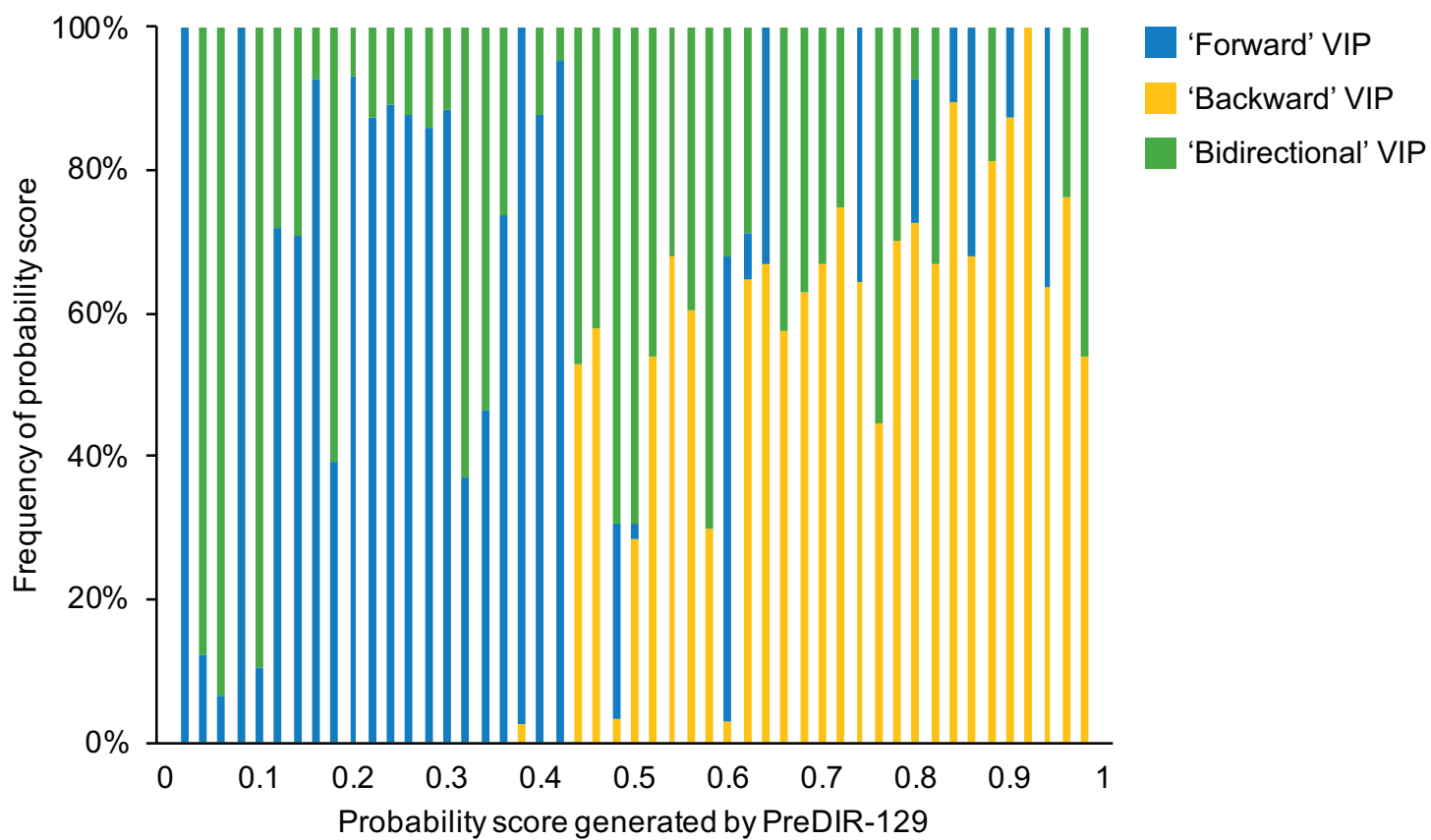

Supplement: S5 Fig — Abbreviations: HIV-1, human immunodeficiency virus type 1; VIPs, HIV-1 interacting human proteins. (PDF) [file pcbi.1009720.s008.pdf]

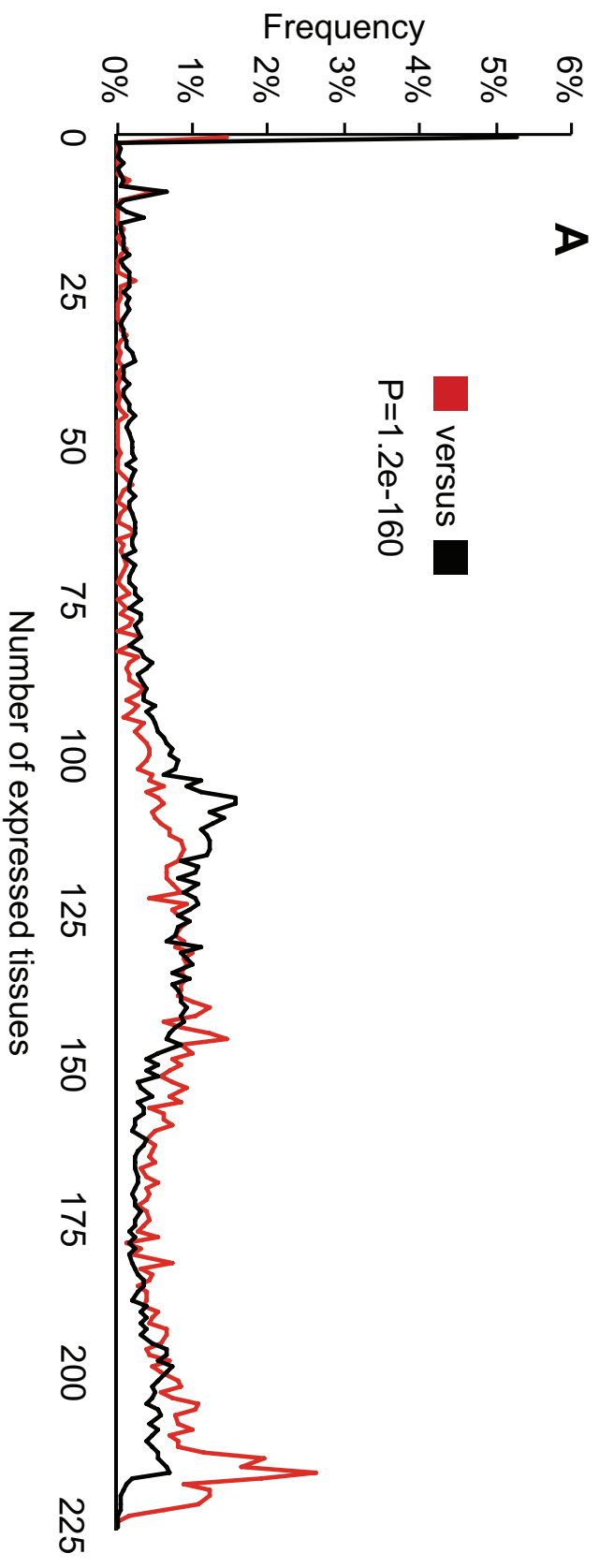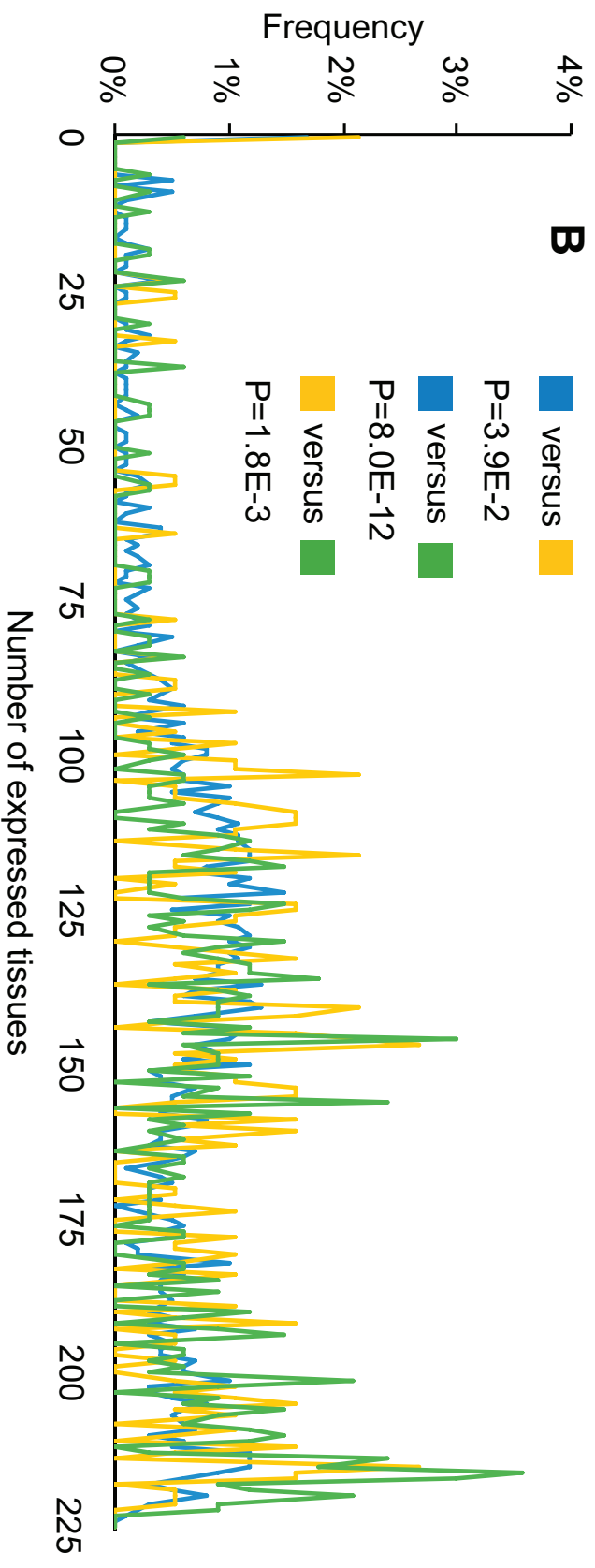

■ VIP   ■ Non-VIP   ■ 'Forward' VIP   ■ 'Backward' VIP   ■ 'Bidirectional' VIP

Supplement: S6 Fig — Abbreviations: HIV-1, human immunodeficiency virus type 1; VIPs, HIV-1 interacting human proteins; non-VIPs, non-HIV-1 interacting human proteins. (PDF) [file pcbi.1009720.s009.pdf]
