# Supplementary material for: In silico prediction of HIV-1-host molecular interactions and their directionality
Source: PLoS Comput Biol. 2022 Feb 8;18(2):e1009720. doi: 10.1371/journal.pcbi.1009720 (PMC8856524; doi:10.1371/journal.pcbi.1009720)
Supplement: S1 Table — (PDF) [file pcbi.1009720.s010.pdf]

S1 Table. The recommended interaction direction for models using the 129 optimum feature set[f].

Score range	Direction distribution ‘backward’/’forward’/’bidirectional’	Recommendation_1[b,c]	Confidence_1[a,d]	Recommendation_2	Confidence_2[e]
[0.00-0.02)	0.00%/0.00%/0.00%	'Forward'	N/A	'Bidirectional'	High
[0.02-0.04)	0.00%/0.00%/0.00%	'Forward'	N/A	'Bidirectional'	High
**[0.04-0.06)**	**0.00%/2.00%/2.09%**	**'Bidirectional'**	**51%**	**'Forward'**	**100.00%**
[0.06-0.08)	0.67%/2.67%/2.69%	'Bidirectional'	45%	'Forward'	88.93%
**[0.08-0.10)**	**0.00%/5.33%/1.49%**	**'Forward'**	**78%**	**'Bidirectional'**	**100.00%**
[0.10-0.12)	0.67%/2.67%/3.28%	'Bidirectional'	50%	'Forward'	89.92%
**[0.12-0.14)**	**0.00%/4.67%/2.69%**	**'Forward'**	**63%**	**'Bidirectional'**	**100.00%**
**[0.14-0.16)**	**0.00%/4.00%/3.88%**	**'Forward'**	**51%**	**'Bidirectional'**	**100.00%**
[0.16-0.18)	0.67%/5.33%/2.09%	'Forward'	66%	'Bidirectional'	91.76%
**[0.18-0.20)**	**0.00%/1.33%/2.69%**	**'Bidirectional'**	**67%**	**'Forward'**	**100.00%**
[0.20-0.22)	0.00%/3.33%/3.28%	'Forward'	50%	'Bidirectional'	100.00%
[0.22-0.24)	2.00%/4.00%/3.28%	'Forward'	43%	'Bidirectional'	78.46%
[0.24-0.26)	0.67%/2.00%/1.49%	'Forward'	48%	'Bidirectional'	83.97%
[0.26-0.28)	1.33%/5.33%/3.58%	'Forward'	52%	'Bidirectional'	86.99%
**[0.28-0.30)**	**0.00%/3.33%/2.69%**	**'Forward'**	**55%**	**'Bidirectional'**	**100.00%**
[0.30-0.32)	0.67%/4.67%/1.49%	'Forward'	68%	'Bidirectional'	90.23%
[0.32-0.34)	1.33%/1.33%/3.28%	'Bidirectional'	55%	'Forward'	77.59%
[0.34-0.36)	0.67%/3.33%/4.48%	'Bidirectional'	53%	'Forward'	92.14%
[0.36-0.38)	0.67%/3.33%/2.69%	'Forward'	50%	'Bidirectional'	90.03%
[0.38-0.40)	2.67%/3.33%/2.09%	'Forward'	41%	'Backward'	74.17%
[0.40-0.42)	1.33%/2.67%/2.09%	'Forward'	44%	'Bidirectional'	78.10%
[0.42-0.44)	1.33%/3.33%/1.79%	'Forward'	52%	'Bidirectional'	79.35%
[0.44-0.46)	2.00%/1.33%/1.49%	'Backward'	41%	'Bidirectional'	72.37%
[0.46-0.48)	2.67%/2.00%/2.09%	'Backward'	39%	'Bidirectional'	70.40%
[0.48-0.50)	0.67%/1.33%/2.09%	'Bidirectional'	51%	'Forward'	83.70%
[0.50-0.52)	2.67%/2.00%/2.99%	'Bidirectional'	39%	'Backward'	73.86%
[0.52-0.54)	5.33%/1.33%/3.58%	'Backward'	52%	'Bidirectional'	86.99%
[0.54-0.56)	3.33%/2.00%/2.09%	'Backward'	45%	'Bidirectional'	73.06%
[0.56-0.58)	2.67%/2.00%/2.39%	'Backward'	38%	'Bidirectional'	71.65%
**[0.58-0.60)**	**1.33%/0.00%/2.69%**	**'Bidirectional'**	**67%**	**'Backward'**	**100.00%**
[0.60-0.62)	2.00%/4.00%/3.58%	'Forward'	42%	'Bidirectional'	79.13%
[0.62-0.64)	2.67%/0.67%/1.79%	'Backward'	52%	'Bidirectional'	86.99%
[0.64-0.66)	2.00%/2.00%/1.79%	'Backward'	35%	'Forward'	69.07%
[0.66-0.68)	6.67%/2.00%/2.99%	'Backward'	57%	'Bidirectional'	82.84%
**[0.68-0.70)**	**6.00%/0.00%/1.19%**	**'Backward'**	**83%**	**'Bidirectional'**	**100.00%**
**[0.70-0.72)**	**1.33%/0.00%/0.60%**	**'Backward'**	**69%**	**'Bidirectional'**	**100.00%**
[0.72-0.74)	2.00%/1.33%/1.49%	'Backward'	41%	'Bidirectional'	72.37%
[0.74-0.76)	2.00%/2.00%/0.60%	'Backward'	44%	'Forward'	87.01%
[0.76-0.78)	2.67%/0.67%/2.99%	'Bidirectional'	47%	'Backward'	89.45%
**[0.78-0.80)**	**3.33%/0.00%/1.79%**	**'Backward'**	**65%**	**'Bidirectional'**	**100.00%**
[0.80-0.82)	2.00%/1.33%/0.90%	'Backward'	47%	'Forward'	78.82%
[0.82-0.84)	3.33%/0.67%/1.19%	'Backward'	64%	'Bidirectional'	87.16%
[0.84-0.86)	2.67%/0.67%/0.30%	'Backward'	73%	'Forward'	91.78%
[0.86-0.88)	2.67%/1.33%/0.90%	'Backward'	54%	'Forward'	81.71%

[0.88-0.90)	5.33%/0.67%/1.19%	'Backward'	74%	'Bidirectional'	90.73%
[0.90-0.92)	3.33%/1.33%/0.90%	'Backward'	60%	'Forward'	83.90%
[0.92-0.94)	4.67%/0.67%/0.90%	'Backward'	75%	'Bidirectional'	89.30%
[0.94-0.96)	1.33%/0.67%/0.30%	'Backward'	58%	'Forward'	87.01%
**[0.96-0.98)**	**4.00%/0.00%/1.49%**	**'Backward'**	**73%**	**'Bidirectional'**	**100.00%**
**[0.98-1.00]**	**4.67%/0.00%/0.60%**	**'Backward'**	**89%**	**'Bidirectional'**	**100.00%**

a: N/A is placed for the lack of reference proteins with predicted prediction scores located within corresponding ranges;

b: recommendation_1 is determined according to the dominant class scored within the corresponding range;

c: in case the dominant class is not obvious, we recommend VIPs with prediction score no less than 0.5 to be 'Backward' and those with prediction score less than 0.5 to be 'Forward';

d: confidence_1 is calculated as the occurrence frequency of dominant class divided by that of all classes;

e: confidence_2 is calculated as the occurrence frequency of the major two classes divided by that of all classes; we can't provide the exact confidence we have when getting a prediction score lower than 0.04, but the tested HIV-1 interacting human protein are very likely to have a 'Forward' or 'Bidirectional' tag;

f: rows are colored as red when we have confidence_1 scored higher than 50%; texts in these rows are further bold when confidence_2 reaches 100%.

Abbreviations: HIV-1, human immunodeficiency virus type 1.
